# Mechanism-based inactivation of human aldehyde oxidase by erlotinib: Mechanistic insights from structural analogs and molecular docking

**DOI:** 10.1016/j.molpha.2025.100097

**Published:** 2025-11-12

**Authors:** Jia Rong Kweh, Nicholas Kai Ming Ng, Le Min Ngoh, Cynthia Jing Yan Li, Bao Jie Tan, Wee Kiat Tan, Vijaya Saradhi Mettu, Karl Austin-Muttitt, Jonathan G.L. Mullins, Aik Jiang Lau

**Affiliations:** 1 Department of Pharmacy, Faculty of Science, National University of Singapore, Singapore, Singapore; 2 Biological Resource Centre, Agency for Science, Technology and Research, Singapore, Singapore; 3 Structural Bioinformatics Group, Faculty of Medicine, Health and Life Science, Swansea University, Swansea, Wales, United Kingdom; 4 College of Pharmacy, Faculty of Health, Dalhousie University, Halifax, Nova Scotia, Canada; 5 Department of Pharmacology, Faculty of Medicine, Dalhousie University, Halifax, Nova Scotia, Canada

**Keywords:** Aldehyde oxidase, Erlotinib, Structural analogs, Mechanism-based inactivation, Molecular docking, Pharmacokinetics

## Abstract

Aldehyde oxidase (AOX1) is a cytosolic molybdo-flavoenzyme that metabolizes azaheterocyclic drugs. Erlotinib and gefitinib are azaheterocyclic drugs. We deployed structural analogs to investigate the molecular interaction between these drugs and AOX1. Erlotinib, *O*-desmethylerlotinib, and *O*-didesmethylerlotinib, but not gefitinib, *O*-desmethylgefitinib, or *O*-desmorpholinopropylgefitinib, decreased carbazeran 4-oxidation by liver cytosol (human, rat, and mouse) and human recombinant AOX1. Erlotinib, *O*-desmethylerlotinib, and *O*-didesmethylerlotinib exhibited time- and concentration-dependent inactivation with unbound inactivation potency (*K*_I,u_) of 1.52, 4.41, and 1.67 *μ*M, respectively. The inactivation was not reversed after dialysis, not protected by nucleophilic trapping agents or scavengers of reactive oxygen species, not affected by an oxidizing or reducing agent, but was attenuated by an alternative AOX1 substrate (*O*^6^-benzylguanine) and competitive AOX1 inhibitor (gefitinib). The terminal alkyne group of erlotinib was essential for AOX1 inactivation, as suggested by the findings for 3-vinylerlotinib (less potent inactivator) and tetrahydroerlotinib (no inactivation). Molecular docking results predicted covalent binding of erlotinib, *O*-desmethylerlotinib, and *O*-didesmethylerlotinib to the molybdenum cofactor. Adding a 4′-methyl group to erlotinib increased the inactivation potency but decreased inactivation efficiency, whereas blocking the C_2_-position of erlotinib with a hydroxy group or a methyl group decreased inactivation potency and efficiency, suggesting that the C_2_-position of erlotinib plays a role in AOX1 inactivation. In mice, erlotinib increased carbazeran (Aox substrate) and decreased 4-oxo-carbazeran (metabolite) levels in blood, liver, and kidneys. Overall, our study provides molecular insights into the mechanism-based inactivation of AOX1 by erlotinib, *O*-desmethylerlotinib, and *O*-didesmethylerlotinib and the irreversible AOX1 inactivation by erlotinib on the pharmacokinetics of AOX1-metabolized drugs.

**Significance Statement:**

This study shows that erlotinib and select metabolites are mechanism-based inactivators of AOX1, provides insights into the mechanism of the inactivation by deploying structural analogs and molecular docking, and demonstrates the in vivo impact on AOX1-mediated drug metabolism.

## 1 Introduction

Aldehyde oxidase (AOX) is a cytosolic molybdo-flavoenzyme that requires a flavin adenine dinucleotide and a molybdenum cofactor for its catalytic activity.^1^ AOX1, the only AOX isoform in humans, has a broad substrate specificity. It oxidizes a wide variety of aldehydes and aromatic heterocycles such as aza-, oxo-, and sulfo-heterocycles.^2^ Drugs such as zaleplon,^3^ methotrexate,^4^ idelalisib,^5^ and endogenous chemicals, such as retinal,^6^ are AOX1 substrates. The catalytic function of AOX1 is subject to reversible, competitive inhibition (eg, by selective estrogen receptor modulators^7^, ^8^, ^9^) and time-dependent inactivation (eg, by hydralazine).^10^^,^^11^ AOX1 inhibition and inactivation have important implications on drug-drug interactions, leading to changes in drug effectiveness or development of adverse drug effects.

A drug may also inhibit a drug metabolizing enzyme by mechanism-based inactivation.^12^^,^^13^ It arises when an enzyme metabolizing a drug generates a reactive intermediate that binds covalently to the enzyme and causes irreversible loss of enzyme activity. The criteria for mechanism-based inactivation include (1) time-dependent inhibition; (2) concentration-dependent inhibition (saturation kinetics); (3) irreversible inactivation; (4) lack of protection of inactivation by catalase and/or superoxide dismutase; (5) protection of inactivation by an alternative substrate or a competitive reversible inhibitor; (6) involvement of a catalytic step or metabolism; and (7) stoichiometry of inactivation.^14^ Clinically, mechanism-based inactivation of drug metabolizing enzymes is a major cause of drug-drug interactions and drug-induced toxicity.^13^

Erlotinib and gefitinib are first-generation epidermal growth factor receptor-tyrosine kinase inhibitors used as first-line therapy for metastatic non-small cell lung cancer.^15^ Both drugs undergo hepatic microsomal CYP450-mediated metabolism.^16^ Erlotinib is metabolized by CYP3A to form *O*-desmethylerlotinib (a major metabolite; also known as OSI-420) and by an unidentified enzyme to *O*-didesmethylerlotinib (minor metabolite),^17^ whereas gefitinib is metabolized by CYP2D6 to *O*-desmethylgefitinib (major metabolite) and by an unidentified enzyme to *O*-desmorpholinopropylgefitinib (minor metabolite).^18^ The chemical structures are shown in Supplemental Fig. 1. Erlotinib and gefitinib are CYP inhibitors.^19^ Gefitinib inhibits CYP2D6,^20^ CYP3A,^21^^,^^22^ and CYP1A1^23^ catalytic activities, whereas erlotinib inhibits CYP3A catalytic activity.^22^ By comparison, erlotinib inhibits UDP-glucuronosyltransferase 1A1 (UGT1A1) enzyme activity, whereas gefitinib inhibits UGT1A1, UGT1A7, UGT1A9, and UGT2B7.^24^ Our previous findings indicate that clinically relevant concentrations of erlotinib, gefitinib, and select in vivo metabolites (*O*-desmethylerlotinib, *O*-didesmethylerlotinib, *O*-desmethylgefitinib, *O*-desmorpholinopropylgefitinib) competitively inhibit the catalytic activity of AOX1.^25^

As a follow-up to our previous study on the competitive inhibition of AOX1 by erlotinib, gefitinib, and select metabolites,^25^ the present study was designed to further investigate the inhibitory interaction of erlotinib, *O*-desmethylerlotinib, *O*-didesmethylerlotinib, gefitinib, *O*-desmethylgeftinib, and *O*-desmorpholinopropylgeftinib on AOX1. In a subsequent study, it was shown that erlotinib is a time-dependent inactivator of AOX1.^26^ However, the biochemical and chemical mechanisms of the time-dependent inactivation of AOX1 by erlotinib remain to be determined. Therefore, the present study was designed to (1) conduct a series of enzymatic experiments and inactivation kinetic analyses and to determine whether erlotinib, gefitinib, and select in vivo metabolites (*O*-desmethylerlotinib, *O*-didesmethylerlotinib, *O*-desmethylgefitinib, and *O*-desmorpholinopropylgefitinib) are mechanism-based inactivators of AOX1; (2) elucidate the chemical mechanism of AOX1 inactivation by deploying structural analogs of erlotinib (Supplemental Fig. 1) as chemical probes and performing molecular docking analyses; and (3) characterize the effect of erlotinib on the in vivo pharmacokinetics of an AOX1 substrate (carbazeran) and metabolite (4-oxo-carbazeran) in mice. Overall, our findings provide insights into the chemical and biochemical mechanisms of AOX1 inactivation by erlotinib and the pharmacokinetic consequences of the irreversible AOX1 inactivation.

## 2 Materials and methods

### 2.1 Chemicals, reagents, and enzymes

Erlotinib, *O*-desmethylerlotinib (OSI-420), *O*-didesmethylerlotinib, 3-vinylerlotinib, tetrahydroerlotinib, 4′-methylerlotinib, *O*-desmethylgefitinib, *O*-desmorpholinopropylgefitinib, carbazeran, 4-oxo-carbazeran (4-hydroxycarbazeran), carbazeran-d4, *O*^6^-benzylguanine, and 8-oxo-*O*^6^-benzylguanine were purchased from Toronto Research Chemicals, Inc. 2-Hydroxyerlotinib and 2-methylerlotinib were custom synthesized by Toronto Research Chemicals, Inc. Gefitinib was bought from Cayman Chemicals. Raloxifene, hydralazine, tolbutamide, L-glutathione (reduced), *N*-acetylcysteine, catalase (> 10,000 U/mg), superoxide dismutase, potassium ferricyanide, and DMSO were purchased from Sigma-Aldrich Corp. Sodium dithionite was bought from Tokyo Chemical Industry Co., Ltd. All other commercially available chemicals were of analytical or high performance liquid chromatographic grade.

Human liver cytosol (pool of 150 donors; equally-mixed sex, catalog #452115, lot #38292, Gentest brand) was purchased from Corning, Inc. Rat liver cytosol (pool of 454 male Sprague-Dawley rats; catalog #R1000.C, lot #1310213) and mouse liver cytosol (pool of 800 male Balb/c mice; catalog #M3000.C, lot #0810496) were purchased from Sekisui XenoTech, LLC. Human recombinant AOX1 enzyme (catalog #CYP150, lot #150011B) and control cytosol (isolated from *Escherichia coli* host cells; catalog #CYP099, lot #INT016E18C) were purchased from Cypex Ltd.

### 2.2 Enzyme inactivation experiments

Carbazeran 4-oxidation and *O*^6^-benzylguanine 8-oxidation were deployed as AOX1-selective catalytic markers.^27^ The assay conditions (amount of cytosolic protein, incubation time, and substrate concentration) for carbazeran 4-oxidation catalyzed by human recombinant AOX1 and human/rat/mouse liver cytosols were optimized previously.^7^^,^^25^ Based on the enzyme kinetics plot,^7^^,^^25^ we selected a high saturating substrate concentration that yielded metabolite formation near *V*_max_. to minimize any competitive inhibition, which was also reduced by the two-step incubation protocol in the AOX1 inactivation assay (below).

Primary incubation mixture (200 *μ*L) containing potassium phosphate buffer (100 mM, pH 7.4) and a test compound (erlotinib, a metabolite, or a structural analog) or DMSO (0.5% v/v; vehicle control) was prewarmed for 3 minutes at 37 °C in a shaking water bath. Enzymatic reaction was initiated by adding human liver cytosol (100 *μ*g). After the specified preincubation duration, an aliquot (10 *μ*L) of the preincubation mixture was transferred to 190 *μ*L of a prewarmed (for 3 minutes at 37 °C) secondary incubation mixture (total volume of 200 *μ*L) containing potassium phosphate buffer and carbazeran (15 *μ*M) or *O*^6^-benzylguanine (150 *μ*M), which yielded a 20-fold dilution of the primary incubation mixture. The secondary incubation mixture was incubated for 5 minutes at 37 °C before the enzymatic reaction was terminated with 200 *μ*L of ice-cold acetonitrile containing carbazeran-d4 (2.5 nM final concentration; internal standard for 4-oxo-carbazeran) or tolbutamide (25 nM final concentration; internal standard for 8-oxo-*O*^6^-benzylguanine). Each sample was immediately mixed by vortex, placed on ice, centrifuged at 16,000*g* for 15 minutes at 4 °C and stored at –30 °C until ultra-high performance liquid chromatography–tandem mass spectrometry (UPLC-MS/MS) analysis. For enzyme inactivation assays involving rat and mouse liver cytosol and recombinant AOX1 assays, the experimental conditions were similar to those of human liver cytosol, except that the amount of cytosolic protein was 200 *μ*g (rat) and 150 *μ*g (mouse), respectively, and the incubation time of the secondary incubation mixture was 60 minutes. For the recombinant AOX1 enzyme assay, the amount of enzyme was 100 *μ*g, the incubation time of the secondary incubation mixture was 15 minutes, and the total volume of the primary and secondary incubation mixtures was reduced to 100 *μ*L.

To determine the enzyme kinetics of AOX1 inactivation by erlotinib and related chemicals, the carbazeran 4-oxidation assay was conducted in the presence of multiple concentrations of an inactivator with various preincubation times, as specified in the figure legend. Preliminary experiments were performed to optimize the incubation time and concentration of an inactivator to ensure a log-linear decrease in enzyme activity over time and that the *k*_obs_ versus inactivator concentration plot reaches a plateau for the determination of the *k*_inact_ value. The observed first-order rate constant for inactivation (*k*_obs_), maximal inactivation rate constant (*k*_inact_), half-maximal inactivation concentration (*K*_I_), and the time required for half of the enzyme molecules to be inactivated (*t*_1/2_) were calculated as described in our previous publication.^28^

### 2.3 Partition ratio determination

The enzyme kinetic experiments were conducted as described above. The incubation was allowed to proceed until saturation of enzyme has been reached (6 or 18 minutes, as specified in each figure legend). Carbazeran 4-oxidation was plotted against the ratio of inactivator concentration to AOX1 protein concentration in the incubation mixture of 200 *μ*L. The specific lot of human liver cytosol (#38292) used in this study contained 1.05 ± 0.01 pmol/*μ*L (mean ± SD) of AOX1 protein, as assessed by capillary nanoproteomic immunoassay.^27^ The turnover number was the extrapolation of the intercept of the 2 linear regression lines to the x-axis, which was determined by solving the 2 linear regression equations. The partition ratio was calculated by subtracting the turnover number by 1, assuming 1:1 stoichiometry between the inactivator and the enzyme.^29^

### 2.4 Rapid equilibrium dialysis and determination of the unbound fraction

Nonspecific binding of test chemicals to human liver cytosol (100 *μ*g) was determined using a Rapid Equilibrium Dialysis Kit (Thermo Fisher Scientific), as described in detail in our previous publication.^27^

### 2.5 Quantification of 4-oxo-carbazeran and 8-oxo-O^6^-benzylguanine by UPLC-MS/MS

The UPLC-MS/MS quantification of 4-oxo-carbazeran, carbazeran, 8-oxo-*O*^6^-benzylguanine, *O*^6^-benzylguanine, and tolbutamide (internal standard for 8-oxo-*O*^6^-benzylguanine) was described in detail in our previous study,^27^ except that carbazeran-d4 was used as an internal standard for the quantification of 4-oxo-carbazeran and carbazeran. Carbazeran-d4 was analyzed using positive electrospray mode and multiple reaction monitoring transitions of mass-to-charge (*m/z*) ratio from 365.3 to 276.1. The compound-dependent MS parameters for carbazeran-d4 were as follows: entrance potential, 10.88 V; collision energy, 27.43 V; collision cell exit potential, 5.2 V; declustering potential, 105.99 V, and dwell time, 200 ms. The MS ion source parameters for carbazeran-d4 were the same as those for 4-oxo-carbazeran and carbazeran. The preparation of the calibration curves for 4-oxo-carbazeran (0.5–1000 nM; equivalent to 0.1–200 pmol) and 8-oxo-*O*^6^-benzylguanine (1–1000 nM; equivalent to 0.2–200 pmol) were described in our previous study.^27^

### 2.6 Quantification of erlotinib, erlotinib metabolites, and analogs by UPLC-MS/MS

Erlotinib, *O*-desmethylerlotinib, *O*-didesmethylerlotinib, and gefitinib (internal standard) were quantified by a UPLC-MS/MS system, as described in our previous study.^25^ For the erlotinib analogs (3-vinylerlotinib, 4′-methylerlotinib, 2-methylerlotinib, and 2-hydroxyerlotinib), the same UPLC conditions were used, and the optimized mass-to-charge (*m/z*) transitions, compound-dependent mass spectrometry parameters, and ion source parameters are shown in Supplemental Table 1.

### 2.7 Effects of exogenous nucleophilic trapping agents and scavengers of reactive oxygen species

An exogenous nucleophilic trapping agent (5 or 10 mM glutathione or *N*-acetylcysteine) or a scavenger of reactive oxygen species (1000 U or 2000 U catalase, or 500 U or 1000 U superoxide dismutase) was preincubated with erlotinib or its metabolites (1.5 *μ*M) and human liver cytosol (100 *μ*g protein) in the primary incubation mixture (200 *μ*L). The control incubations were described in our previous publication.^28^ Each standard incubation mixture was subjected to the same procedures as described in *Enzyme Inactivation Experiments*. The residual AOX1 enzyme activity was determined by the carbazeran 4-oxidation assay.

### 2.8 Effects of a competing AOX1 substrate and a competitive AOX1 inhibitor

*O*^6^-Benzylguanine (AOX1 substrate)^27^ or gefitinib (AOX1 competitive inhibitor)^25^ was preincubated with erlotinib (1.5 *μ*M), *O*-desmethylerlotinib (1.5 *μ*M), *O*-didesmethylerlotinib (1.5 *μ*M), 3-vinylerlotinib (10 *μ*M), 4′-methylerlotinib (3 *μ*M), or 2-methylerlotinib (1 or 2 *μ*M), and human liver cytosol (100 *μ*g protein) in the primary incubation mixture (200 *μ*L). The molar ratio of *O*^6^-benzylguanine (120 and 240 *μ*M) to erlotinib, *O*-desmethylerlotinib, or *O*-didesmethylerlotinib (1.5 *μ*M) was 80 and 160, respectively. The molar ratio of *O*^6^-benzylguanine (120, 240, and 480 *μ*M) to 3-vinylerlotinib (10 *μ*M) was 12, 24, and 48, *O*^6^-benzylguanine to 4′-methylerlotinib (3 *μ*M) was 40, 80, and 160, and *O*^6^-benzylguanine to 2-methylerlotinib (1 *μ*M) was 120, 240, and 480, respectively. The final concentrations of gefitinib were 3, 10 and 30 *μ*M, corresponding to a molar ratio of gefitinib to erlotinib, *O*-desmethylerlotinib, or *O*-didesmethylerlotinib (1.5 *μ*M) of 2, 6.67, and 20, gefitinib to 3-vinylerlotinib (10 *μ*M) of 0.3, 1, and 3, gefitinib to 4′-methylerlotinib (3 *μ*M) of 1, 3.33, and 10, and gefitinib to 2-methylerlotinib (2 *μ*M) of 1.5, 5, and 15, respectively. Each standard incubation mixture was subjected to the same procedures as described in *Enzyme Inactivation Experiments*. The residual AOX1 enzyme activity was determined by the carbazeran 4-oxidation or *O*^6^-benzylguanine 8-oxidation assay.

### 2.9 Dialysis experiment

Primary incubation mixture (200 *μ*L) containing erlotinib, *O*-desmethylerlotinib, *O*-didesmethylerlotinib (1.5 *μ*M) or DMSO (0.5% v/v; vehicle), and potassium phosphate buffer (100 mM, pH 7.4) was prewarmed for 3 minutes at 37 °C in a shaking water bath. Enzymatic reaction was initiated by adding human liver cytosol (100 *μ*g protein), and the mixture was preincubated for 0 or 6 minutes. Subsequently, an aliquot (10 *μ*L) of primary incubation mixture was transferred to 190 *μ*L of prewarmed secondary incubation mixture (200 *μ*L) containing potassium phosphate buffer and carbazeran (15 *μ*M). Simultaneously, another aliquot (150 *μ*L) of the primary incubation mixture was transferred to a Slide-A-Lyzer mini dialysis device (0.1 mL) with a molecular weight cutoff of 10,000 (Pierce Chemical Co.) and placed in a beaker filled with 300 mL of ice-cold potassium phosphate buffer (100 mM, pH 7.4). Additionally, potassium phosphate buffer (300 mL) was replaced at 2 and 4 hours after the start of dialysis. Dialysis was performed at 4 °C for 4 and 24 hours with constant gentle stirring. Subsequently, 10 *μ*L of the dialyzed mixture was transferred to the secondary incubation mixture and the residual AOX1 enzyme activity was determined by the carbazeran 4-oxidation assay.

### 2.10 Effects of an oxidizing and a reducing agent

#### 2.10.1 Preincubation with an oxidizing or reducing agent

Potassium ferricyanide (5 mM; oxidizing agent), sodium dithionite (5 mM; reducing agent), or potassium phosphate buffer (vehicle for oxidizing/reducing agent) was preincubated with human liver cytosol (200 *μ*g protein) in a total volume of 100 *μ*L at 37 °C for 10 minutes. After 10-minute incubation, an aliquot (50 *μ*L) of the primary preincubation mixture was transferred to 150 *μ*L of a prewarmed secondary incubation mixture (total volume of 200 *μ*L) containing erlotinib (1.5 *μ*M), 2-methylerlotinib (2 *μ*M), 2-hydroxyerlotinib (2 *μ*M), or vehicle control (DMSO, 0.5% v/v) at 37 °C for 0 or 6 minutes, which yielded a 4-fold dilution of the primary incubation mixture. After a 0 or 6-minute preincubation duration, an aliquot (10 *μ*L) of the secondary incubation mixture was transferred to a prewarmed tertiary incubation mixture (total volume of 200 *μ*L) containing potassium phosphate buffer and carbazeran (15 *μ*M), which yielded a 20-fold dilution of the secondary incubation mixture. The tertiary incubation mixture was incubated for 5 minutes at 37 °C before the enzymatic reaction was terminated with 200 *μ*L of ice-cold acetonitrile containing carbazeran-d4 (2.5 nM final concentration; internal standard for 4-oxo-carbazeran). The residual AOX1 enzyme activity was determined by the carbazeran 4-oxidation assay.

#### 2.10.2 Coincubation with an oxidizing agent

Erlotinib (1.5 *μ*M), *O*-desmethylerlotinib (1.5 *μ*M), *O*-didesmethylerlotinib (1.5 *μ*M), 3-vinylerlotinib (10 *μ*M), tetrahydroerlotinib (10 *μ*M), 4′-methylerlotinib (3 *μ*M), 2-methylerlotinib (2 *μ*M), hydralazine (10 *μ*M; positive control),^10^ gefitinib (10 *μ*M; negative control),^25^ or vehicle control (DMSO, 0.5% v/v) was coincubated with human liver cytosol (100 *μ*g protein) in the absence or presence of potassium ferricyanide (2 mM) at 37 °C for 0 or 6 minutes. The residual AOX1 enzyme activity was determined by the carbazeran 4-oxidation assay.

#### 2.10.3 Postincubation with an oxidizing agent

A test chemical at a concentration described above was incubated with human liver cytosol (100 *μ*g protein) at 37 °C for 0 or 6 minutes. An aliquot (20 *μ*L) of the preincubation mixture was transferred to a prewarmed secondary incubation mixture (total volume of 100 *μ*L) containing potassium ferricyanide (2 mM) or vehicle control (potassium phosphate buffer) at 37 °C for 10 minutes, which yielded a 5-fold dilution of the primary incubation mixture. After 10-minute incubation, an aliquot (50 *μ*L) of the secondary incubation mixture was transferred to a prewarmed secondary incubation mixture (total volume of 200 *μ*L) containing potassium phosphate buffer and carbazeran (15 *μ*M), which yielded a 4-fold dilution of the secondary incubation mixture. The residual AOX1 enzyme activity was determined as described above.

### 2.11 Molecular docking

Molecular docking was performed as described previously.^7^^,^^25^ To investigate covalent bonding potentials, it was necessary to consider bond dissociation energies, as the Protein-Ligand ANT System software program does not contain the potential values to simulate the tight-binding interactions exhibited by these compounds by default. The bond dissociation energies, estimates of the activation and reaction energies, were informed by use of the tables in “Bond Dissociation Energies.”^30^

For erlotinib and its metabolites, the triple bond was changed to a double bond to simulate the formation of the intermediate, except for 3-vinylerlotinib, in which the double bond was changed to a single bond. For erlotinib, and for its metabolites with an intact triple bond, a linear potential was then added to Protein-Ligand ANT System that imparts the C-S bond energy, –170 kcal/mol, if the active carbon and active sulfur are in direct contact. The potential decays to 0, stepwise, over a separation distance of 1.5 Å. Furthermore, the simulated overall energy for those ligands had a constant of +53 kcal/mol added, to account for the difference between the bond energy and the estimated net energy of the reaction, ie, the term ensured the calculated binding energy correctly reflected the estimated net reaction energy of –117 kcal/mol. For 3-vinylerlotinib, the constant was +81 kcal/mol, to reflect a net energy of –89 kcal/mol. To simulate the docking of hydralazine, preparation of the protein with a missing oxygen atom in the molybdenum cofactor was generated. A similar linear potential was added that imparts the Mo…N coordination energy, –92 kcal/mol, when the cofactor Mo and active N were in contact. A constant of +90 kcal/mol was added to the scoring function, to reflect the estimated net energy of –2 kcal/mol.

### 2.12 Pharmacokinetics study in mice

BALB/c mice (male, ∼8 weeks old) were purchased and housed at the Biological Resource Centre, Agency for Science, Technology and Research (A∗STAR). The experimental protocol was approved by the A∗STAR Institutional Review Board. Carbazeran and erlotinib were suspended and diluted with 40% v/v polyethylene glycol (PEG-400; vehicle) to 1 and 10 mg/mL, respectively. The mice were predosed orally with either erlotinib (100 mg/kg) or PEG-400 (40% v/v; vehicle) prior to the start of treatment with carbazeran. At 2 hours after erlotinib or vehicle pretreatment, both groups of mice were orally administered with carbazeran (10 mg/kg p.o.). Blood (12.5 *μ*L) was drawn from the tail vein (with a needle prick) at various times (0.25, 0.5, 0.75, 1, 2, 3, 4, 6, and 8 hours) after carbazeran dosing. The blood samples were diluted 2 times with 12.5 *μ*L of water. Acetonitrile containing the internal standard (carbazeran-d4, 4 nM in 75 *μ*L acetonitrile and 3 nM in final sample volume of 100 *μ*L) was added to the blood samples to precipitate proteins. The diluted blood samples (total volume of 100 *μ*L) were mixed thoroughly, centrifuged at 16,000*g* for 15 minutes at 4 °C, and stored at –30 °C until UPLC-MS/MS analysis. Blank and standard calibration samples were prepared from control mice that were not treated with any chemicals. Pharmacokinetic data were analyzed by noncompartmental analysis using Phoenix WinNonlin 6.3 (Certara).

At the end of the last blood collection (8 hour post-carbazeran dosing), the mice were sacrificed. The liver and kidney tissues were harvested and weighed, and Tris (0.05 M)–KCl (1.15%) buffer (pH 7.5) solution (2 times the weight of tissues) was added. The tissue samples were homogenized, and a 12.5 *μ*L aliquot of the homogenate was diluted with an equal volume of water. Acetonitrile containing the internal standard (carbazeran-d4, 4 nM in 75 *μ*L acetonitrile and 3 nM in final sample volume of 100 *μ*L) was added to the diluted tissue samples (25 *μ*L) to precipitate the proteins. The diluted tissue samples (total volume of 100 *μ*L) were mixed, centrifuged at 16,000*g* for 15 minutes at 4 °C, and stored at –30 °C until UPLC-MS/MS analysis.

### 2.13 Statistical analysis

Data were analyzed by one-way or two-way analysis of variance and, where appropriate, were followed by the Student-Newman-Keuls multiple comparison test (SigmaPlot 12.5). Data obtained from experiments with 2 groups were analyzed by the Student’s *t* test (one-tail). The level of statistical significance was set a priori at *P* < .05. Data are expressed as mean ± SD. The present study was an exploratory study, and the *P* values generated in present study were not to be interpreted as hypothesis testing, but as descriptive information.

## 3 Results

### 3.1 Differential time-dependent inactivation of human recombinant AOX1 and hepatic cytosolic AOX1 (human, rat, and mouse) by erlotinib, gefitinib, and select in vivo metabolites

**Fig. 1:**
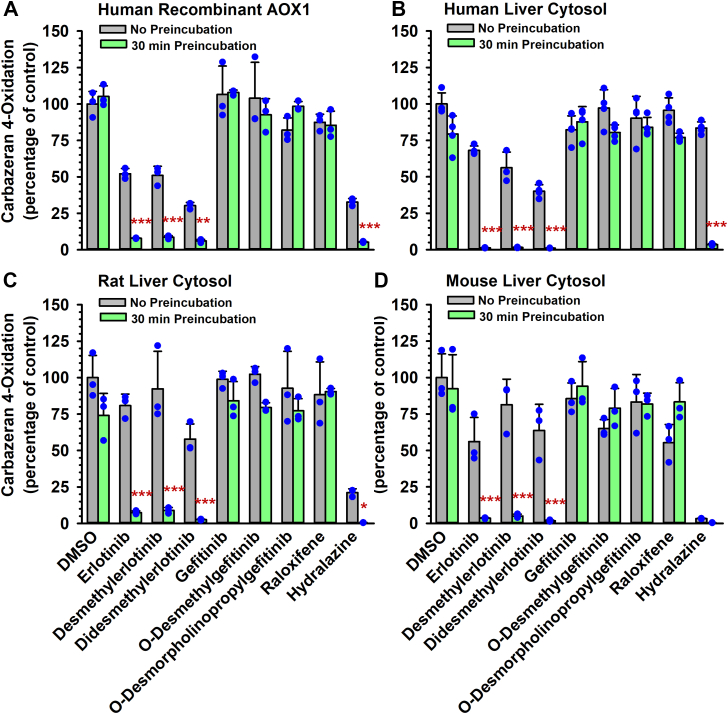
Effect of preincubation of erlotinib, *O*-desmethylerlotinib, *O*-didesmethylerlotinib, gefitinib, *O*-desmethylgefitinib, and *O*-desmorpholinopropylgefitinib with human recombinant AOX1 or human/rodent liver cytosols on carbazeran 4-oxidation. (A) Human recombinant AOX1 (100 *μ*g protein), (B) human liver cytosol (100 *μ*g protein), (C) rat liver cytosol (200 *μ*g protein), or (D) mouse liver cytosol (150 *μ*g protein) was preincubated with erlotinib, *O*-desmethylerlotinib, *O*-didesmethylerlotinib, gefitinib, *O*-desmethylgefitinib, *O*-desmorpholinopropylgefitinib (each at 10 *μ*M), hydralazine (10 *μ*M; positive control), raloxifene (0.02 *μ*M; negative control), or vehicle control (DMSO, 0.5% v/v) at 37 °C for 0 or 30 minutes. An aliquot (10 *μ*L) of the primary incubation mixture was transferred to a secondary incubation mixture containing carbazeran (15 *μ*M) and incubated for (A) 180, (B) 5, or (C, D) 120 minutes. Data are expressed as percentage of activity in the vehicle-treated control group that was not subjected to preincubation and expressed as mean ± SD of 3 independent experiments. *∗P* < .05, *∗∗P* < .01, ∗∗∗*P* < .001, significantly different from (1) the vehicle-treated control group subjected to 30-minute preincubation and (2) the same treatment group that was not subjected to preincubation.

Preincubation of erlotinib, *O*-desmethylerlotinib, or *O*-didesmethylerlotinib (Supplemental Fig. 1) with human recombinant AOX1 for 30 minutes decreased carbazeran 4-oxidation by 92%, 91%, and 94%, as compared with the vehicle control group that was not subjected to preincubation (Fig. 1A), respectively. A similar pattern was evident when an alternate AOX-1 substrate (*O*^6^-benzylguanine)^27^ was used in the enzyme inactivation assay (Supplemental Fig. 2). Conversely, gefitinib, *O*-desmethylgefitinib, and *O*-desmorpholinopropylgefitinib (Supplemental Fig. 1) did not decrease carbazeran 4-oxidation after 30-minute preincubation with the enzyme (Fig. 1A). Preincubation of gefitinib with human liver cytosol for a longer time (120 minutes) still did not decrease carbazeran 4-oxidation (data not shown).

Given the species differences in AOX expression^31^ and in the extent of competitive inhibition of AOX1 catalytic activity,^25^^,^^32^ we compared the effect of the drugs and metabolites on the inactivation of AOX1-catalyzed carbazeran 4-oxidation by pooled human, rat, or mouse liver cytosols, which express AOX1.^1^ After 30-minute preincubation of the drugs with a liver cytosol, erlotinib, *O*-desmethylerlotinib, and *O*-didesmethylerlotinib decreased carbazeran 4-oxidation by 99% in human liver cytosol (Fig. 1B), 92%–97% in rat liver cytosol (Fig. 1C), and 95%–98% in mouse liver cytosol (Fig. 1D), whereas gefitinib and select in vivo metabolites did not decrease the activity. As expected, raloxifene, which is a negative control for time-dependent AOX1 inactivation,^9^ did not decrease carbazeran 4-oxidation after a 30-minute preincubation with AOX1 enzyme, whereas hydralazine, a positive control for time-dependent AOX1 inactivation,^10^ decreased the activity in the in vitro models (Fig. 1, A–D).

### 3.2 Kinetics of AOX1 inactivation by erlotinib, O-desmethylerlotinib, and O-didesmethylerlotinib

**Fig. 2:**
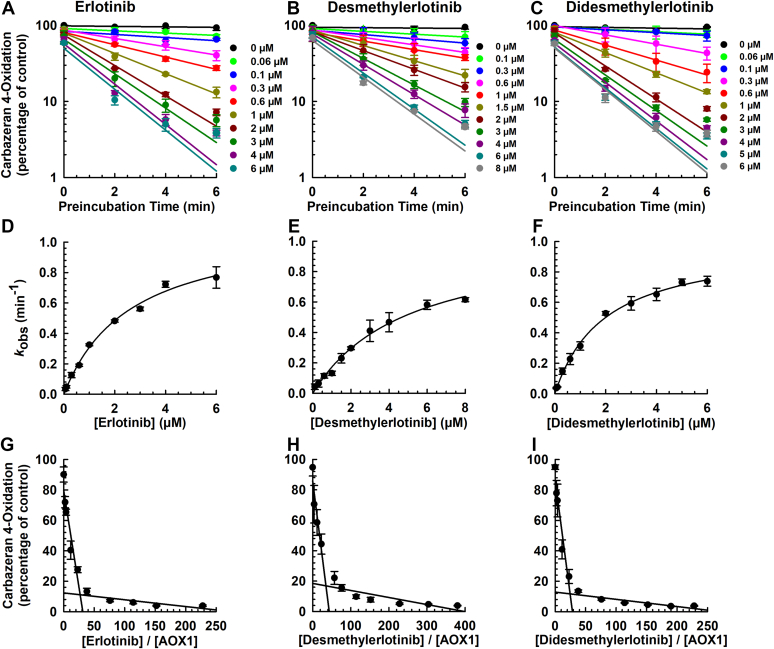
Time- and concentration-dependent inactivation of AOX1 by erlotinib, *O*-desmethylerlotinib, and *O*-didesmethylerlotinib. Human liver cytosol (100 *μ*g protein) was preincubated with (A, D, G) erlotinib (0.06, 0.1, 0.3, 0.6, 1, 2, 3, 4, or 6 *μ*M), (B, E, H) *O*-desmethylerlotinib (0.1, 0.3, 0.6, 1, 1.5, 2, 3, 4, 6, or 8 *μ*M), (C, F, I) *O*-didesmethylerlotinib (0.06, 0.1, 0.3, 0.6, 1, 2, 3, 4, 5, or 6 *μ*M), or vehicle control (DMSO, 0.5% v/v) at 37 °C for 0, 2, 4, or 6 minutes. An aliquot (10 *μ*L) of the primary incubation mixture was transferred to a secondary incubation mixture containing carbazeran (15 *μ*M). Data are expressed as percentage of activity in the vehicle-treated control group that was not subjected to preincubation and expressed as mean ± SD of 3 independent experiments. (A, B, C) Plot of log of AOX1 activity (carbazeran 4-oxidation) remaining against preincubation time for erlotinib, *O*-desmethylerlotinib, and *O*-didesmethylerlotinib. Values of observed inactivation rate constant (*k*_obs_) were determined from the initial slopes of each line. (D, E, F) A nonlinear regression plot of *k*_obs_ versus concentration of erlotinib, *O*-desmethylerlotinib, and *O*-didesmethylerlotinib. (G, H, I) Partition ratio plot for the inactivation of AOX1 by erlotinib, *O*-desmethylerlotinib, and *O*-didesmethylerlotinib. The turnover number was determined from the point of intersection to the abscissa, and the partition ratio was determined by subtracting the turnover number by 1.

To determine the inactivation kinetics of erlotinib, *O*-desmethylerlotinib, and *O*-didesmethylerlotinib, the inactivation experiments were carried out at 37 °C for 0, 2, 4, and 6 minutes with various concentrations of inactivator. Erlotinib (Fig. 2A), *O*-desmethylerlotinb (Fig. 2B), and *O*-didesmethylerlotinib (Fig. 2C) inactivated AOX1 in a time- and concentration-dependent manner. Nonlinear regression plots of observed first-order rate constant for inactivation (*k*_obs_) values against inactivator concentrations (Fig. 2, D–F) were constructed to determine the inactivation kinetic parameters, which were compared to the in vivo concentrations in humans^33^, ^34^, ^35^ (Table 1). The maximal inactivation rate constant (*k*_inact_) values for erlotinib, *O*-desmethylerlotinib, and *O*-didesmethylerlotinib were comparable, whereas the inactivator concentration at half-maximal rate of inactivation (*K*_I_) values, which are inversely proportional to inactivation potency, were in the rank order of *O*-desmethylerlotinib > erlotinib > *O*-didesmethylerlotinib (Table 1).

**Table 1:** Enzyme inactivation of human liver cytosolic AOX1-mediated carbazeran 4-oxidation by erlotinib, select metabolites, and structural analogs Shown are unbound inactivation constant (*K*_I,u_), *k*_inact_, *k*_inact_/*K*_I_, t_1/2,_ turnover number, partition ratio, maximum plasma drug concentrations (C_max_), and unbound maximum plasma drug concentrations (C_max,u_). Experimental data are expressed as mean ± SD for 3 independent experiments

Chemical	*k*_inact_ (min^–1^)	*K*_I_ (*μ*M)	*K*_I,u_ (*μ*M)^a^	*k*_inact_/*K*_I_ (min^–1^*μ*M^–1^)	Inactivation t_1/2_ (min)	Turnover Number	Partition Ratio	C_max_ (*μ*M)	C_max,u_ (*μ*M)^e^
Erlotinib	1.14 ± 0.17	2.73 ± 0.73	1.52 ± 0.41	0.43 ± 0.05	0.62 ± 0.09	26.9 ± 1.30	25.9 ± 1.30	4.41–5.39^b^	0.31–0.38
*O*-Desmethylerlotinib	1.05 ± 0.06	5.23 ± 1.24	4.41 ± 1.04	0.21 ± 0.04∗∗∗	0.66 ± 0.04	35.2 ± 4.78	34.2 ± 4.78	0.11–0.26^c^	0.0077–0.02
*O*-Didesmethylerlotinib	1.00 ± 0.04	1.98 ± 0.20	1.67 ± 0.17	0.50 ± 0.03∗∗	0.70 ± 0.03	24.6 ± 1.29	23.6 ± 1.29	0.002–0.011^d^	0.0001–0.0008
3-Vinylerlotinib	0.33 ± 0.07∗∗∗	30.6 ± 9.60∗∗∗	23.9 ± 7.53∗∗∗	0.01 + 0.00∗∗∗	2.14 ± 0.47∗	271 ± 23.5∗∗∗	270 ± 23.5∗∗∗	N/A	N/A
4′-Methylerlotinib	0.14 ± 0.03∗∗∗	1.03 ± 0.27	0.75 ± 0.20	0.13 ± 0.02∗∗∗	5.29 ± 1.19∗∗∗	42.9 ± 4.32	41.9 ± 4.32	N/A	N/A
2-Methylerlotinib	0.84 ± 0.10∗∗	4.00 ± 0.27	2.04 ± 0.14	0.21 ± 0.01∗∗∗	0.83 ± 0.11	49.9 ± 1.40	48.9 ± 1.40	N/A	N/A
2-Hydroxyerlotinib	0.25 ± 0.03∗∗∗	23.9 ± 3.68∗∗∗	18.7 ± 2.87∗∗∗	0.01 ± 0.00∗∗∗	2.80 ± 0.34∗∗∗	392 ± 0.82∗∗∗	391 ± 0.82∗∗∗	N/A	N/A
Tetrahydroerlotinib	No inactivation							N/A	N/A

N/A, human data not available; n.d., not determined or no data.

∗*P* < .05, ∗∗*P* < .01, ∗∗∗*P* < .001, significantly different from the parent drug group.

a *K*_I,u_ = f_u_ × *K*_I_, where f_u_ was calculated from 10 *μ*M of chemicals in human liver cytosol (Supplemental Table 1).

b For cancer patients administered with 150 mg/day of erlotinib, the C_max_ was 1737 ng/mL (4.41 *μ*M) on day 24 and 2120 ng/mL (5.39 *μ*M) on day 28.^33^

c For healthy non-smoker volunteers administered with a single 150 mg dose of erlotinib, the C_max_ was 43.5–98.3 ng/mL (0.11–0.26 *μ*M).^34^

d For cancer patients administered with 150 mg/day of erlotinib for 1–2 months, the C_24h_ was 0.86–4.2 ng/mL (2.35–11.49 nM).^35^

e C_max,u_ = plasma f_u_ × C_max_. Plasma f_u_ = 0.07, based on erlotinib (TARCEVA), package insert, 2016. For *O*-desmethylerlotinib and *O*-didesmethylerlotinib, the f_u_ is assumed to be similar to the parent drug.

Compared with erlotinib (the parent drug), the *k*_inact_/*K*_I_ ratio, an indicator of the efficiency of enzyme inactivation,^36^ was lower for *O*-desmethylerlotinib, and higher for *O*-didesmethylerlotinib. The time required for the inactivation of half of the enzyme molecules (t_1/2_) was comparable for erlotinib and its 2 metabolites. Equilibrium dialysis (4-hour dialysis time) was performed to investigate the extent of nonspecific binding of the chemicals to human liver cytosol (100 *μ*g protein). As shown in Supplemental Table 2, the fraction unbound values (f_u_) for erlotinib, *O*-desmethylerlotinib, and *O*-didesmethylerlotinib were 0.56, 0.84, and 0.84, respectively. After correcting for the nonspecific protein binding, the unbound *K*_I_ values (*K*_I,u_) for erlotinib, *O*-desmethylerlotinib, and *O*-didesmethylerlotinib were in the low micromolar range of 1.52, 4.41, and 1.67 *μ*M, respectively (Table 1).

Partition ratio denotes the number of inactivator molecules metabolized for every molecule of enzyme inactivated, reflecting the efficiency of the inactivation.^14^^,^^29^ The respective partition ratios were determined using a titration method, whereby increasing concentrations of inactivator were added to a constant amount of AOX1. As shown in Table 1, the turnover number and partition ratio were comparable for erlotinib, *O*-desmethylerlotinib, and *O*-didesmethylerlotinib.

### 3.3 Role of the terminal alkyne group of erlotinib in AOX1 inactivation

**Fig. 3:**
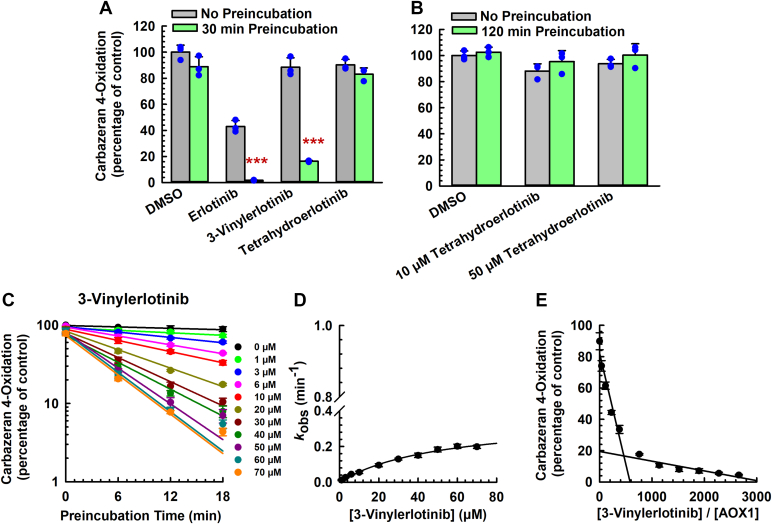
Comparative effect of erlotinib, 3-vinylerlotinib, and tetrahydroerlotinib on the inactivation of AOX1. (A) Human liver cytosol (100 *μ*g protein) was preincubated with erlotinib (10 *μ*M), 3-vinylerlotinib (10 *μ*M), tetrahydroerlotinib (10 *μ*M), or vehicle control (DMSO, 0.5% v/v) at 37 °C for 0 or 30 minutes. (B) Human liver cytosol (100 *μ*g protein) was preincubated with tetrahydroerlotinib (10 or 50 *μ*M) or vehicle control (DMSO, 0.5% v/v) at 37 °C for 0 or 120 minutes. (C–E) Human liver cytosol (100 *μ*g protein) was preincubated with 3-vinylerlotinib (1, 3, 6, 10, 20, 30, 40, 50, 60, or 70 *μ*M), or vehicle control (DMSO, 0.5% v/v) at 37 °C for 0, 6, 12, or 18 minutes. An aliquot (10 *μ*L) of the primary incubation mixture was transferred to a secondary incubation mixture containing carbazeran (15 *μ*M). Data are expressed as percentage of activity in the vehicle-treated control group that was not subjected to preincubation and expressed as mean ± SD of 3 independent experiments. (C) Plot of log of AOX1 activity (carbazeran 4-oxidation) remaining against preincubation time for 3-vinylerlotinib. Values of observed inactivation rate constant (*k*_obs_) were determined from the initial slopes of each line. (D) A nonlinear regression plot of *k*_obs_ versus concentration of 3-vinylerlotinib. (E) Partition ratio plot for the inactivation of AOX1 by 3-vinylerlotinib. The turnover number was determined from the point of intersection to the abscissa, and the partition ratio was determined by subtracting the turnover number by 1. ∗∗∗*P* < .001, significantly different from (1) the vehicle-treated control group subjected to 30-minute preincubation and (2) the same treatment group that was not subjected to preincubation.

Given that erlotinib, but not gefitinib, inactivated AOX1 (Fig. 1), we hypothesized that the terminal alkyne group of erlotinib is responsible for the inactivation. To test this experimental hypothesis, we compared the effects of 2 structural analogs, 3-vinylerlotinib (double bond, Supplemental Fig. 1) and tetrahydroerlotinib (single bond, Supplemental Fig. 1), with that of erlotinib (triple bond, Supplemental Fig. 1). Carbazeran 4-oxidation was decreased by 98% by erlotinib and 84% by 3-vinylerlotinib (Fig. 3A), whereas tetrahydroerlotinib at 10 *μ*M for 30 minutes (Fig. 3A) or 50 *μ*M for 120 minutes (Fig. 3B) did not decrease the activity.

3-Vinylerlotinib did not decrease carbazeran 4-oxidation after 6 minutes of preincubation (data not shown), which was a contrast to erlotinib (Fig. 1). Given that 3-vinylerlotinib inactivated AOX1 activity after 30 minutes but not after 6-minute preincubation, we postulated that the kinetics of inactivation between erlotinib and 3-vinylerlotinib are different. We then determined the kinetics of 3-vinylerlotinib with longer time points up to 30 and 120 minutes (data not shown), but the activity did not decrease linearly at some concentrations. Therefore, in our final optimized assay on 3-vinylerlotinib, we performed the kinetics experiment up to 18 minutes (3-fold longer than that used for erlotinib). Similar to erlotinib, 3-vinylerlotinib decreased carbazeran 4-oxidation in a time- and concentration-dependent manner within 18 minutes (Fig. 3, C and D). As shown in Table 1, the *k*_inact_, *K*_I_, *k*_inact_/*K*_I_ ratio, and t_1/2_ value for 3-vinylerlotinib were approximately 3.5-fold lower, 11-fold higher, 43-fold lower, and 3.5-fold longer, when compared with erlotinib, respectively. In addition, the turnover number and partition ratio for AOX1 inactivation by 3-vinylerlotinib were 271 and 270 (Fig. 3E; Table 1), respectively, which were 10-fold greater than those of erlotinib.

### 3.4 Role of the 4- and C_2_-position of erlotinib in AOX1 inactivation

**Fig. 4:**
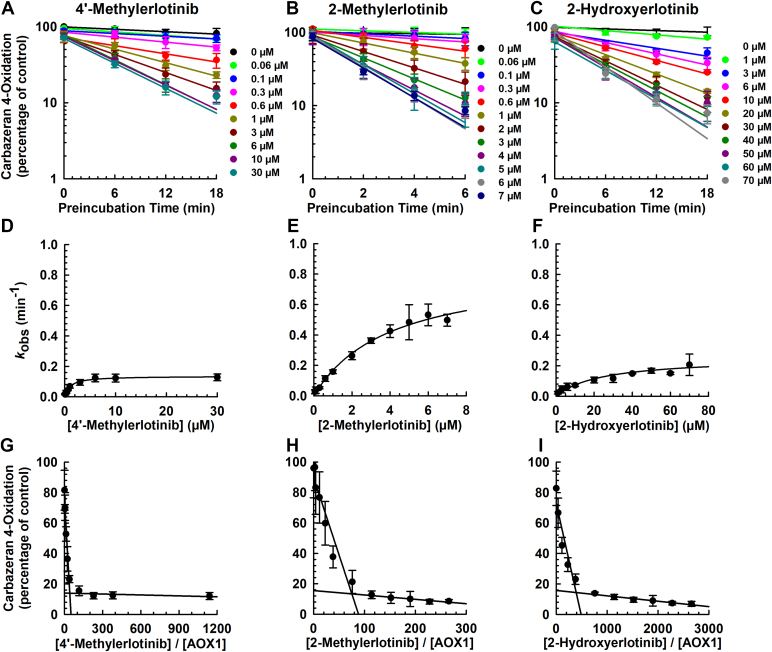
Time- and concentration-dependent inactivation of AOX1 by erlotinib structural analogs (4′-methylerlotinib, 2-methylerlotinib, and 2-hydroxyerlotinib). Human liver cytosol (100 *μ*g protein) was preincubated with (A, D, G) 4′-methylerlotinib (0.06, 0.1, 0.3, 0.6, 1, 3, 6, 10, or 30 *μ*M), (B, E, H) 2-methylerlotinib (0.06. 0.1, 0.3, 0.6, 1, 2, 3, 4, 5, 6, or 7 *μ*M), (C, F, I) 2-hydroxyerlotinib (1, 3, 6, 10, 20, 30, 40, 50, 60, or 70 *μ*M), or vehicle control (DMSO, 0.5% v/v) at 37 °C for (A, C) 0, 6, 12, or 18 minutes or (B) 0, 2, 4, or 6 minutes. An aliquot (10 *μ*L) of the primary incubation mixture was transferred to a secondary incubation mixture containing carbazeran (15 *μ*M). Data are expressed as percentage of activity in the vehicle-treated control group that was not subjected to preincubation and expressed as mean ± SD of 3 independent experiments. (A, B, C) Plot of log of AOX1 activity (carbazeran 4-oxidation) remaining against preincubation time for 4′-methylerlotinib, 2-methylerlotinib, and 2-hydroxyerlotinib. Values of observed inactivation rate constant (*k*_obs_) were determined from the initial slopes of each line. (D, E, F) A nonlinear regression plot of *k*_obs_ versus concentration of 4′-methylerlotinib, 2-methylerlotinib, and 2-hydroxyerlotinib. (G, H, I) Partition ratio plot for the inactivation of AOX1 by 4′-methylerlotinib, 2-methylerlotinib, and 2-hydroxyerlotinib. The turnover number was determined from the point of intersection to the abscissa, and the partition ratio was determined by subtracting the turnover number by 1.

Given that the alkyne group of erlotinib played a key role in AOX1 inactivation, our next research question was whether the 4-position of erlotinib, which is adjacent to the alkyne group, hinders or increases the access of the molecule to AOX1. To answer this question, we compared the inactivation kinetic profiles of 4′-methylerlotinib (Supplemental Fig. 1) with that of erlotinib (parent drug). Preliminary experiments were performed to optimize the preincubation duration of the chemical with human liver cytosol, so that a log-linear decrease in enzyme activity was obtained. Varying concentrations of 4′-methylerlotinib were then preincubated with human liver cytosol for the optimized duration as specified in Fig. 4. AOX1 inactivation by 4′-methylerlotinib occurred in a time- and concentration-dependent manner (Fig. 4A). Nonlinear regression plot of *k*_obs_ against inactivator concentration (Fig. 4D) was constructed to determine the corresponding inactivation parameters. As shown in Table 1, the *k*_inact_, *K*_I_, *k*_inact_/*K*_I_ ratio, and t_1/2_ value for 4′-methylerlotinib were decreased by 88%, 62%, 70%, and 8.5-fold longer, when compared with erlotinib, respectively. The turnover number and partition ratio (Fig. 4G) for AOX1 inactivation by 4′-methylerlotinib were 1.6-fold greater than those of erlotinib. These findings suggest that the 4-position of erlotinib, which is adjacent to the alkyne group, is associated with increased potency and decreased rate of AOX1 inactivation.

Based on the chemical structure of erlotinib and the structure-metabolism relationship of AOX1,^37^ we hypothesized that C_2_-metabolites/analogs of erlotinib may also be mechanism-based inactivator of AOX1. Therefore, we custom synthesized 2 structural analogs of erlotinib, one with a methyl (2-methylerlotinib, Supplemental Fig. 1) and the other one with a hydroxyl group (2-hydroxyerlotinib, Supplemental Fig. 1; a proposed metabolite of erlotinib). 2-Methylerlotinib (Fig. 4B) and 2-hydroxyerlotinib (Fig. 4C) exhibited time- and concentration-dependent inactivation of AOX1 activity. Nonlinear regression plots of *k*_obs_ versus inactivation concentration (Fig. 4, E and F) showed that the kinetics reached saturation. As shown in Table 1, the *k*_inact_ value of 2-methylerltoinib was 26% and 2-hydroxyerlotinib was 78% lesser than that of erlotinib. The *K*_I_ value of 2-methylerltoinib was 1.5-fold and 2-hydroxyerlotinib was 8.9-fold greater than that of erlotinib. The *k*_inact_/*K*_I_ ratio of 2-methylerltoinib was 51% and 2-hydroxyerlotinib was 98% lesser than that of erlotinib. The t_1/2_ value of 2-methylerltoinib was 1.3-fold and 2-hydroxyerlotinib was 4.6-fold longer than that of erlotinib. The partition ratio for AOX1 inactivation by 2-methylerlotinib and 2-hydroxyerlotinib (Fig. 4, H and I) was 1.9-fold and 15.4-fold greater than those of erlotinib. Overall, these findings suggest that the C_2_ position of erlotinib plays a role in the AOX1 inactivation by erlotinib.

### 3.5 Molecular docking of erlotinib and select metabolites/analogs to the active site of AOX1

**Fig. 5:**
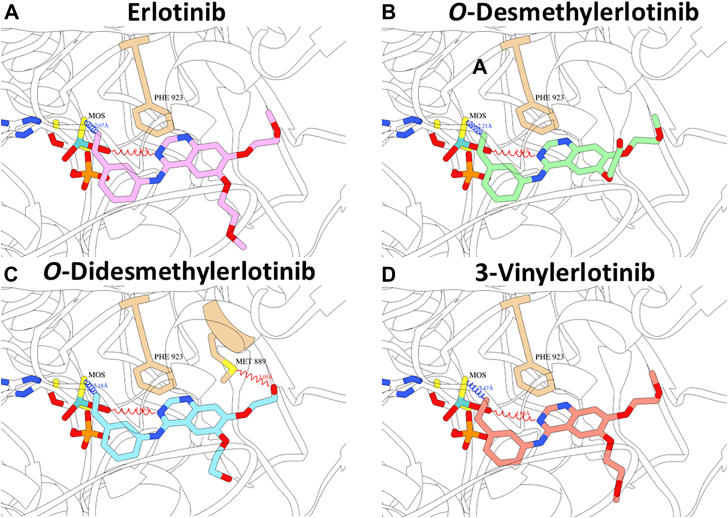
Molecular docking of erlotinib and select metabolites and analogs to the active site of human AOX1. The predicted binding of the compounds is shown with the molybdenum cofactor MOS visible toward the center of each frame. Shown are the key residues and the key interactions of (A) erlotinib, (B) *O*-desmethylerlotinib, (C) *O*-didesmethylerlotinib, and (D) 3-vinylerlotinb with human AOX1.

Molecular docking analysis was performed to provide additional insight to our experimental data on AOX1 inactivation by erlotinib and select metabolites and analogs. Structural modeling of the compounds within the AOX1 pocket shows that erlotinib, *O*-desmethylerlotinb, and *O*-didesmethylerlotinib bound in a similar orientation (Fig. 5, A–C). The interacting moiety for erlotinib was the alkyne group, which targets thiol groups present in active sites containing cysteine residues.^38^ A proposed mode of interaction is a thiol-yne reaction between alkyne group of erlotinib (R-C≡C) and dioxothiomolybdenum (VI) ion (MOS) sulfur (HS-R) to form a vinyl group (R-C=C-S-R). For erlotinib (Fig. 5A), the alkyne group covalently bound to the MOS sulfur, and the central ring was coordinated by an H-bond with a MOS oxygen and by π-stacking with Phe-923. The carbonyl group on the sidechain was not able to form a bond with Met 889. For *O*-desmethylerlotinib (Fig. 5B), the interactions with MOS and Phe-923 were maintained. However, the shorter sidechain possessed weaker van der Waals interactions than the carbonyl form found in erlotinib, and in this orientation adopted a less stable configuration, which slightly reduced the affinity of interaction of the ligand. For *O*-didesmethylerlotinib (Fig. 5C), the carbon atom of the alkyne group covalently bound to sulfur in the molybdenum-oxygen-sulfur complex. The central nitrogen-containing ring H-bonds to the molybdenum cofactor was also coordinated by Phe-923 by a π-stacking interaction. Finally, the hydroxyl group in the truncated (with respect to erlotinib) sidechain was able to form a unique H-bond with Met-889, explaining the greater affinity of *O*-didesmethylerlotinib to AOX1.

When compared with erlotinib (Fig. 5A), 3-vinylerlotinib (Fig. 5D) showed weaker interactions because it coordinated a H-bond with the molybdenum cofactor less effectively, and its intermediate bound with the active carbon further away from the cofactor thiol group (Table 2). In terms of the key interactions of 3-vinylerlotinib, the ethyl intermediate of the vinyl group caused the ligand to have a lower potency than erlotinib. This was not only because of the higher activation energy for the covalent bond with the MOS sulfur, but because the different bond angles in the ligand caused its central ring to bind further away from the MOS oxygen, thereby weakening that H-bond. The distance of the covalent bond with MOS was longer, and the binding efficiency was lesser than those of erlotinib and its metabolites (Table 2).

**Table 2:** Molecular docking analysis of human AOX1 inactivators *K*_I_ values were determined as described in Materials and Methods

Inactivator	Distance to MOS-S (Covalent Bond) (Å)	Distance to MOS-O (H-Bond) (Å)	Other key Residues and Interactions	Simulated Binding Efficiency (kcal/mol/Da)	Experimental Log *K*_I_ (*μ*M)
Erlotinib	2.07	3.71	Phe-923 (pi)	–0.48	0.43 ± 0.11
*O*-Desmethylerlotinib	2.21	3.79	Phe-923 (pi)	–0.49	0.71 ± 0.11∗
*O*-Didesmethylerlotinib	2.18	3.88	Phe-923 (pi), Met-889 (H-bond, 3.05 Å)	–0.51	0.30 ± 0.04
3-Vinylerlotinib	2.47	3.98	Phe-923 (pi)	–0.40	1.5 ± 0.15∗∗∗
Hydralazine	MOS (Mo-N)	Nil	Phe-885 (pi), Phe-923 (pi)	–0.37	n.d.

∗*P* < .05, ∗∗∗*P* < .001, significantly different from the parent drug (erlotinib) group. Experimental data are expressed as mean ± SD for 3 independent experiments.

### 3.6 Irreversible AOX1 inactivation by erlotinib, O-desmethylerlotinib, and O-didesmethylerlotinib

In the remaining inactivation experiments (sections *Irreversible AOX1 Inactivation by Erlotinib, O*-*Desmethylerlotinib, and O*-*Didesmethylerlotinib, Lack of Protective Effect by Exogenous Nucleophilic Trapping Agents and Scavengers of Reactive Oxygen Species on AOX1 Inactivation by Erlotinib, O*-*Desmethylerlotinib, and O*-*Didesmethylerlotinib, An Alternate AOX1 Substrate and An AOX1 Competitive Inhibitor Attenuated AOX1 Inactivation by Erlotinib, Metabolites, and Structural Analogs, and Lack of Effect of an Oxidizing Agent or a Reducing Agent on AOX1 Inactivation by Erlotinib, Select Metabolites, and Structural Analogs*), the concentrations of the drug and metabolites selected were based on the inactivation kinetics data, whereby the concentrations were within the linear range (ie, nonsaturating concentration) of the *k*_obs_ versus inactivator concentration plot to ensure sensitivity to modulation by dialysis or other reagents.

**Fig. 6:**
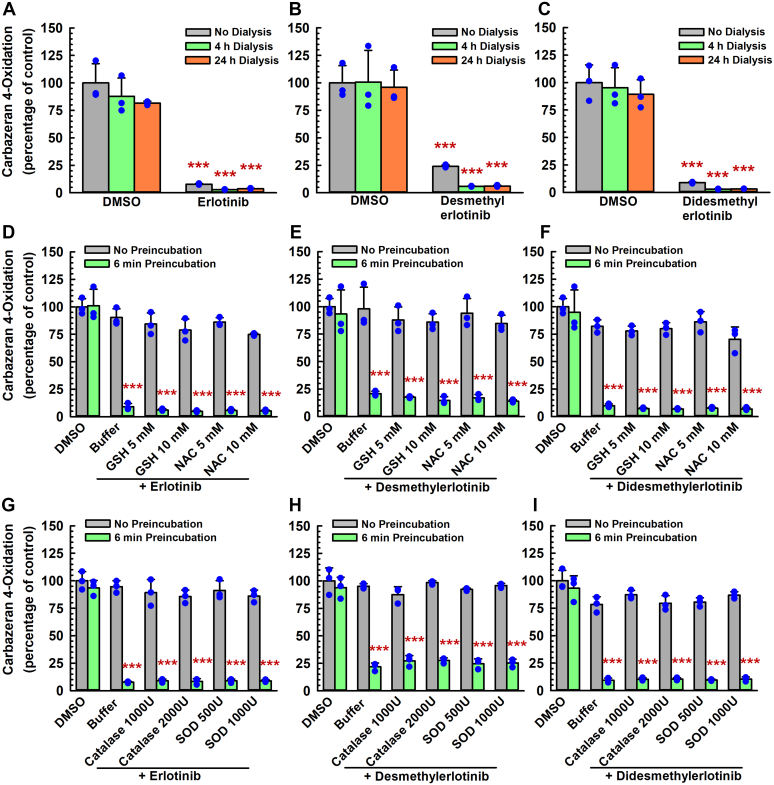
Effect of dialysis, nucleophilic trapping agents, and scavengers of reactive oxygen species on inactivation of liver cytosolic AOX1 by erlotinib, *O*-desmethylerlotinib, and *O*-didesmethylerlotinib. (A–C) Human liver cytosol (100 *μ*g protein) was preincubated with erlotinib (1.5 *μ*M), *O*-desmethylerlotinib (1.5 *μ*M), *O*-didesmethylerlotinib (1.5 *μ*M), or vehicle control (DMSO, 0.5% v/v) at 37 °C for 0 or 6 minutes. An aliquot (10 *μ*L) of the primary incubation mixture was transferred directly to a secondary incubation mixture containing carbazeran (15 *μ*M), whereas an aliquot (150 *μ*L) was subjected to 4 or 24 hours of dialysis at 4 °C before an aliquot (10 *μ*L) of the dialyzed sample was transferred to the secondary incubation mixture. Data are expressed as percentage of activity in the nondialyzed vehicle-treated control group after 6-minute preincubation and expressed as mean ± SD of 3 independent experiments. ∗∗∗*P* < .001, significantly different from the vehicle-treated control group subjected to 6-minute preincubation without dialysis, 4-hour dialysis, or 24-hour dialysis. (D–I) Human liver cytosol (100 *μ*g protein) was preincubated with erlotinib (1.5 *μ*M), *O*-desmethylerlotinib (1.5 *μ*M), *O*-didesmethylerlotinib (1.5 *μ*M), or vehicle control (DMSO, 0.5% v/v) and (D–F) glutathione (5 or 10 mM), (D–F) *N*-acetylcysteine (5 or 10 mM), (G–I) catalase (1000 or 2000 U), or (G–I) superoxide dismutase (500 or 1000 U) at 37 °C for 0 or 6 minutes. An aliquot (10 *μ*L) of the primary incubation mixture was transferred to a secondary incubation mixture containing carbazeran (15 *μ*M). Data are expressed as percentage of activity in the vehicle-treated control group that was not subjected to preincubation and expressed as mean ± SD of 3 independent experiments. ∗∗∗*P* < .001, significantly different from (1) the vehicle-treated control group subjected to 6-minute preincubation and (2) the same treatment group that was not subjected to preincubation.

To determine whether the inactivation of AOX1 by erlotinib and its metabolites is irreversible, human liver cytosol was preincubated with the drug or a metabolite and the primary incubation mixture was subjected to dialysis at 4 °C. As shown in Fig. 6, A–C, carbazeran 4-oxidation activity was not recovered after 4 and 24 hours of dialysis. The residual AOX1 activities were 7.8% (without dialysis) and 3.7% (24-hour dialysis) of vehicle-treated control (nondialyzed) for the erlotinib-treated group (Fig. 6A). Similarly, the inactivation of AOX1 activity by *O*-desmethylerlotinib (Fig. 6B) and *O*-didesmethylerlotinib (Fig. 6C) was not reversed after 4 or 24 hours of dialysis, indicating irreversibility of the inactivation. The residual activity of the vehicle-treated control group did not decrease after 4 or 24 hours of dialysis (Fig. 6, A–C), indicating that the dialysis process did not decrease the enzyme activity.

### 3.7 Lack of protective effect by exogenous nucleophilic trapping agents and scavengers of reactive oxygen species on AOX1 inactivation by erlotinib, O-desmethylerlotinib, and O-didesmethylerlotinib

To determine whether exogenous nucleophilic trapping agents or scavengers of reactive oxygen species have a protective effect on AOX1 inactivation by erlotinib, *O*-desmethylerlotinib, and *O*-didesmethylerlotinib, we compared the remaining AOX1 activities of the primary incubations in the presence or absence of glutathione, *N*-acetylcysteine, catalase, or superoxide dismutase. As shown in Fig. 6, D–F, erlotinib, *O*-desmethylerlotinib, and *O*-didesmethylerlotinib decreased carbazeran 4-oxidation by 90%, 69%, and 90% after 6-minute preincubation with human liver cytosol, respectively, but the addition of glutathione (5 and 10 mM) and *N*-acetylcysteine (5 and 10 mM) to the primary incubation mixture did not reduce the inactivation by erlotinib (Fig. 6D), *O*-desmethylerlotinib (Fig 6E), and *O*-didesmethylerlotinib (Fig. 6F). Correspondingly, catalase (1000 and 2000 U) and superoxide dismutase (500 and 1000 U) did not protect AOX1 inactivation by erlotinib (Fig. 6G), *O*-desmethylerlotinib (Fig. 6H), or *O*-didesmethylerlotinib (Fig. 6I). These results indicate that AOX1 inactivation by erlotinib, *O*-desmethylerlotinib, and *O*-didesmethylerlotinib was confined to the active site, the inactivation occurred prior to release of the inactivating species, and that hydrogen peroxide and other reactive oxygen species were not involved in the inactivation.

### 3.8 An alternate AOX1 substrate and an AOX1 competitive inhibitor attenuated AOX1 inactivation by erlotinib, metabolites, and structural analogs

**Fig. 7:**
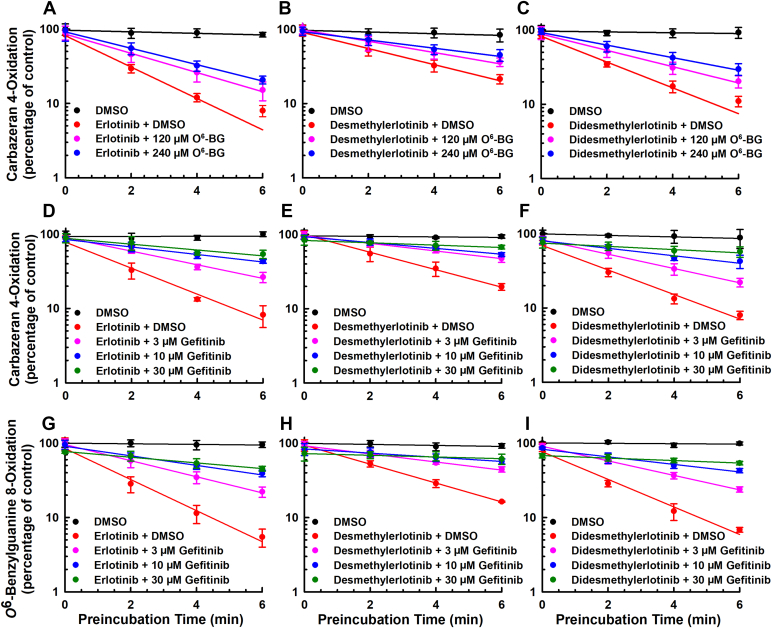
Effect of an alternative AOX1 substrate (*O*^6^-benzylguanine) and a competitive inhibitor (gefitinib) on inactivation of human liver cytosolic carbazeran 4-oxidation and *O*^6^-benzylguanine 8-oxidation by erlotinib, *O*-desmethyerlotinib, and *O*-didesmethylerlotinib. Human liver cytosol (100 *μ*g protein) was preincubated with (A) erlotinib (1.5 *μ*M), (B) *O*-desmethylerlotinib (1.5 *μ*M), (C) *O*-didesmethylerlotinib (1.5 *μ*M), or vehicle control (DMSO, 0.5% v/v) in the absence or presence of *O*^6^-benzylguanine (120 or 240 *μ*M) at 37 °C for 0, 2, 4, or 6 minutes. Human liver cytosol (100 *μ*g protein) was preincubated with (D, G) erlotinib (1.5 *μ*M), (E, H) *O*-desmethylerlotinib (1.5 *μ*M), (F, I) *O*-didesmethylerlotinib (1.5 *μ*M), or vehicle control (DMSO, 0.5% v/v) in the absence or presence of gefitinib (3, 10, or 30 *μ*M) at 37 °C for 0, 2, 4, or 6 minutes. An aliquot (10 *μ*L) of the primary incubation mixture was transferred to a secondary incubation mixture containing carbazeran (15 *μ*M) or *O*^6^-benzylguanine (150 *μ*M). Data are expressed as percentage of activity in the vehicle-treated control group that was not subjected to preincubation and expressed as mean ± SD of 3 independent experiments.

To examine whether the inactivation of AOX1 by erlotinib, *O*-desmethylerlotinib, and *O*-didesmethylerlotinib occurs at the active site of the enzyme, *O*^6-^benzylguanine (a selective AOX1 substrate)^27^ was preincubated with an inactivator and human liver cytosol, at 37 °C for 6 minutes, and the remaining AOX1 activity was compared. The molar ratio of *O*^6-^benzylguanine (120 and 240 *μ*M) to an inactivator (1.5 *μ*M) was 80 and 160, respectively, because our enzyme kinetics experiment showed that *O*^6-^benzylguanine substrate has a high *K*_m_ value.^27^ Erlotinib (Fig. 7A), *O*-desmethylerlotinib (Fig. 7B), and *O*-didesmethylerlotinib (Fig. 7C) decreased carbazeran 4-oxidation by 92%, 79%, and 89%, respectively, and *O*^6-^benzylguanine (120 and 240 *μ*M) attenuated the extent of the inactivation in a concentration-dependent manner. The control experiments included primary incubation mixture (1) without both *O*^6-^benzylguanine and inactivator (vehicle-treated control) and that (2) without inactivator (ie, *O*^6-^benzylguanine alone). All the controls yielded the expected results. *O*^6-^benzylguanine, at 120 and 240 *μ*M, alone did not affect the activity (data not shown).

Next, we confirmed the results that the inactivators bind to the active site of AOX1 by using an AOX1 inhibitor, gefitinib, that inhibits AOX1 by a reversible competitive mechanism at the active site^25^ and did not show time-dependent inactivation (Fig. 1). As shown by Fig. 7, D–F, erlotinib, *O*-desmethylerlotinib, or *O*-didesmethylerlotinib alone, in the absence of gefitinib, decreased carbazeran 4-oxidation by 91%, 80%, and 92%, respectively. In the presence of 3, 10 and 30 *μ*M gefitinib, erlotinib decreased the activity by 73%, 55%, and 47%, respectively (Fig. 7D). Increasing concentrations of gefitinib attenuated the inactivation by *O*-desmethylerlotinib (Fig. 7E) and *O*-didesmethylerlotinib (Fig. 7F) in a similar pattern. As expected, there was no decrease in activity for the vehicle-treated control or gefitinib alone group at the various time points. To confirm that substrate differences would not affect the conclusion, we repeated the experiment using *O*^6-^benzylguanine as a substrate in the secondary incubation to determine the remaining AOX1 activity after the human liver cytosol was preincubated with an inactivator. Gefitinib attenuated the extent of AOX1 inactivation by erlotinib (Fig. 7G), *O*-desmethylerlotinib (Fig. 7H) and *O*-didesmethylerlotinib (Fig. 7I) in a similar manner.

Similar experiments were performed on the structural analogs of erlotinib that showed AOX1 inactivation. As shown in Supplemental Fig. 3, A–C, *O*^6-^benzylguanine (120, 240, and 480 *μ*M) attenuated the extent of AOX1 inactivation by 3-vinylerlotinib, 4′-methylerlotinib, and 2-methylerlotinib in a concentration-dependent manner. In addition, gefitinib (3, 10, and 30 *μ*M) attenuated the extent of AOX1 inactivation by 3-vinylerlotinib, 4′-methylerlotinib, and 2-methylerlotinib in a concentration-dependent manner when carbazeran 4-oxidation (Supplemental Fig. 4, A–C) or *O*^6-^benzylguanine 8-oxidation was determined (Supplemental Fig. 4D).

### 3.9 Lack of effect of an oxidizing agent or a reducing agent on AOX1 inactivation by erlotinib, select metabolites, and structural analogs

#### 3.9.1 Preincubation with an oxidizing or reducing agent

**Fig. 8:**
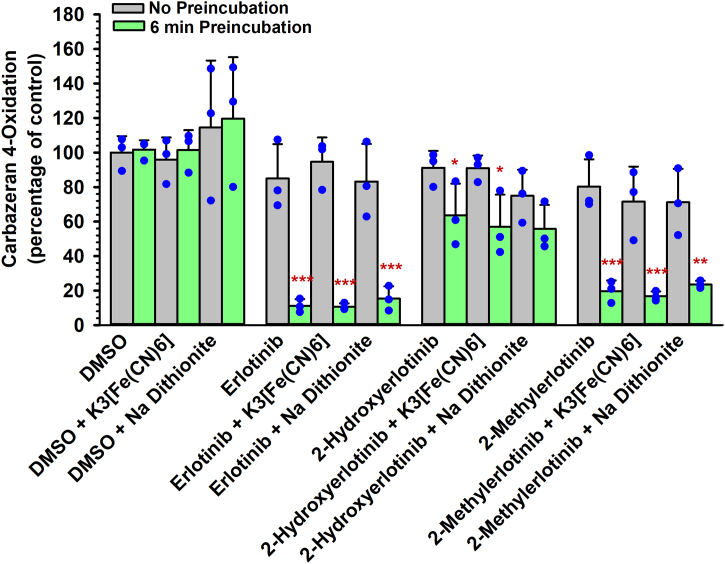
Effect of an oxidizing agent (potassium ferricyanide) and a reducing agent (sodium dithionite) on the inactivation of human liver cytosolic carbazeran 4-oxidation by erlotinib, 2-hydroxyerlotinib, and 2-methyerlotinib. Human liver cytosol (100 *μ*g protein) was first preincubated with either potassium ferricyanide (5 mM), sodium dithionite (5 mM), or buffer at 37 °C for 10 minutes. An aliquot (50 *μ*L) of the primary incubation mixture was transferred to a secondary incubation mixture containing erlotinib (1.5 *μ*M), 2-hydroxyerlotinib (2 *μ*M), 2-methylerlotinib (2 *μ*M), or vehicle control (DMSO, 0.5% v/v) at 37 °C for 0 or 6 minutes. Subsequently, an aliquot (10 *μ*L) of the secondary incubation mixture was transferred to a tertiary incubation mixture containing carbazeran (15 *μ*M). Data are expressed as percentage of activity in the vehicle-treated control group that was not subjected to preincubation and expressed as mean ± SD of 3 independent experiments. *∗P* < .05, *∗∗P* < .01, ∗∗∗*P* < .001, significantly different from (1) the vehicle-treated control group subjected to 6-minute preincubation and (2) the same treatment group that was not subjected to preincubation.

AOX1 preferentially catalyzes oxidation reactions of azaheterocyclic rings at electron-deficient positions.^37^ Based on the chemical structure of erlotinib, the C_2_-position of erlotinib was postulated to be oxidized by AOX1, whereas the Mo(VI) cofactor of AOX1 will be reduced to Mo(IV) during the oxidation process. To determine whether oxidizing the Mo cofactor of AOX1 decreases the inactivation of AOX1 by erlotinib, 2-methylerlotinib, and 2-hydroxyerlotinib, we preincubated human liver cytosol with an oxidizing agent, potassium ferricyanide. To determine whether reducing the Mo cofactor of AOX1 enhances the inactivation of AOX1 by erlotinib, 2-methylerlotinib, and 2-hydroxyerlotinib, we preincubated human liver cytosol with a reducing agent, sodium dithionite, prior to incubating the enzyme with the test chemical. As shown in Fig. 8, neither sodium dithionite nor potassium ferricyanide affected the extent of AOX1 inactivation by erlotinib, 2-methylerlotinib, and 2-hydroxyerlotinib. These results indicated that premodifying the oxidative state of AOX1 prior to the addition of the inactivators did not influence AOX1 inactivation, suggesting that metabolism of the inactivator was needed to convert the Mo cofactor to the reduced state.

### 3.9.2 Coincubation and postincubation with an oxidizing agent

To further determine whether an oxidizing agent reverses the inactivation of AOX1 by erlotinib, select metabolites, and structural analogs, we tested the effect of coincubating potassium ferricyanide with erlotinib, *O*-desmethylerlotinib, *O*-didesmethylerlotinib, 3-vinylerlotinib, tetrahydroerlotinib, 4′-methylerlotinib, 2-methyerlotinib, hydralazine (positive control),^10^ or gefitinib (negative control).^25^ As shown in Supplemental Fig. 5, in the assays involving coincubating or postincubating an AOX1 inactivator with an oxidizing agent, the inactivation of AOX1 by erlotinib, *O*-desmethylerlotinib, *O*-didesmethylerlotinib, 3-vinylerlotinib, 4′-methylerlotinib, 2-methylerlotinib, and hydralazine was not increased or decreased by potassium ferricyanide, and the lack of effect of tetrahydroerlotinib and gefitinib (negative control) was not changed by the presence of the oxidizing agent. These results further confirm that AOX1 inactivation by erlotinib, select metabolites, and structural analogs was irreversible, and an oxidizing agent, whether in competition with an AOX1 inactivator (coincubation assay) or added after the incubation with an AOX1 inactivator (postincubation assay), was not able to displace the inactivators from the enzyme.

### 3.10 Erlotinib modulated the pharmacokinetics of carbazeran and 4-oxo-carbazeran in mice

**Fig. 9:**
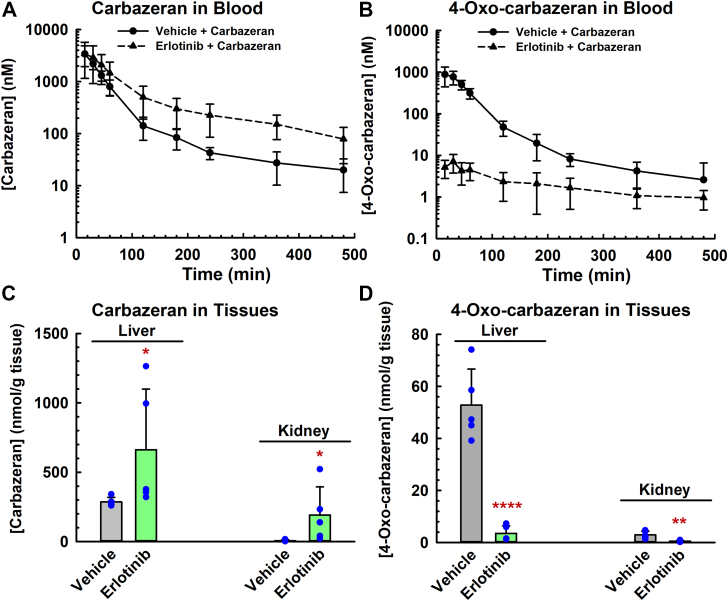
Effect of erlotinib on the pharmacokinetics of carbazeran and 4-oxo-carbazeran in mice. The mice were administered with either erlotinib (100 mg/kg, p.o.) or PEG-400 (40% v/v; vehicle) at 2 hours prior to dosing with carbazeran (10 mg/kg p.o.). Blood (12.5 *μ*L) was drawn from the tail vein at various times (0.25, 0.5, 0.75, 1, 2, 3, 4, 6, and 8 hours) post-carbazeran dosing. After the last time point, the mice were sacrificed, and the liver and kidneys were harvested. The amount of carbazeran and 4-oxo-carbazeran present in the whole blood (A, B), liver (C, D), and kidney (C, D) samples was quantified using UPLC-MS/MS. Data are expressed as mean ± SD for 7 (A, B) or 5 mice (C, D). *∗P* < .05, *∗∗P* < .01, ∗∗∗∗*P* < .0001, significantly different from the parent drug group.

To corroborate our in vitro findings that erlotinib is a potent inactivator of AOX1, we conducted an in vivo pharmacokinetic study in mice. Mice were chosen because mouse and human liver cytosols were inactivated by the chemicals in the same pattern (Fig. 1). Mice were pretreated orally with either erlotinib (100 mg/kg) or PEG-400 (40% v/v; vehicle) for 2 hours prior to the start of treatment with carbazeran. As compared with the vehicle-treated control group, the mice pretreated with erlotinib showed a greater concentration of carbazeran (Fig. 9A) and a far lesser concentration of the 4-oxo-carbazeran metabolite in the blood samples at all the time points (Fig. 9B). Erlotinib increased the concentration of carbazeran in liver and kidneys at 8 hours after administration of carbazeran (Fig. 9C), whereas erlotinib decreased the concentration of 4-oxo-carbazeran metabolite in the tissues (Fig. 9D). Table 3 shows that erlotinib impaired the elimination pharmacokinetics of carbazeran (as shown by the larger area under the curve [AUC], decreased oral clearance, and longer mean residence time), whereas erlotinib had no effect on the absorption pharmacokinetics of carbazeran (as shown by lack of an effect on T_max_ and C_max_). The AUC (metabolite)/AUC (parent) ratio for the control and erlotinib-treated groups was 0.31 and 0.006, respectively, thereby showing a 98% decrease in drug exposure after erlotinib treatment and indicating that the metabolism of carbazeran (Aox substrate) was decreased to a near maximal extent over the course of the blood sampling period (8 hours) and time after erlotinib administration (10 hours), which is equivalent to 14 elimination half-lives of erlotinib in mice.^39^

**Table 3:** Effect of erlotinib on the pharmacokinetic parameters of carbazeran and 4-oxo-carbazeran in mice Mice were pretreated orally with erlotinib 2 hours prior to the oral dosing of carbazeran, and blood was drawn from the tail vein as described in the legend of Fig. 9. Data were analyzed by noncompartmental analysis and expressed as mean ± SD for 7 mice in each group

		Carbazeran		4-Oxo-Carbazeran	
Parameter	Units	Control	Erlotinib	Control	Erlotinib
T_max_	h	0.29 ± 0.09	0.29 ± 0.09	0.32 ± 0.12	0.46 ± 0.27
C_max_	*μ*M	3.47 ± 1.39	3.53 ± 2.18	0.92 ± 0.39	0.008 ± 0.003∗∗∗∗
AUC_0-∞_	*μ*M.h	2.52 ± 0.46	4.60 ± 2.02∗	0.78 ± 0.23	0.027 ± 0.009∗∗∗∗
Ratio of AUC_0-∞_ (metabolite) to AUC_0-∞_ (parent)		N/A	N/A	0.31 ± 0.06	0.007 ± 0.003∗∗∗∗
Apparent oral clearance (CL/F)	L/h/kg	11.3 ± 1.92	6.82 ± 2.23∗∗∗	N/A	N/A
MRT_0-∞_	h	1.32 ± 0.44	2.32 ± 0.76∗∗	0.98 ± 0.19	8.98 ± 8.73∗

AUC_0-∞_, area under the drug concentration-time curve; MRT_0-∞_, mean residence time; N/A, not applicable; T_max_, time to reach maximum drug concentration.

∗ *P* < .05, ∗∗*P* < .01, ∗∗∗*P* < .001, *∗∗∗P* < .0001, significantly different from the control group (one-tailed Student’s *t* test).

## 4 Discussion

A novel finding in the present study is that erlotinib and select metabolites (*O*-desmethylerlotinib and *O*-didesmethylerlotinib) are mechanism-based inactivators of AOX1. This conclusion is based on the following experimental findings: (1) time-dependent inactivation of AOX1 (Fig. 1, Fig. 2, Fig. 3, Fig. 4; Supplemental Fig. 2); (2) concentration-dependent inactivation with saturation inactivation kinetics (Fig. 2, Fig. 3, Fig. 4); (3) irreversible inactivation of AOX1 after 4 and 24 hours of dialysis (Fig. 6, A–C) and postincubation with potassium ferricyanide (Supplemental Fig. 5); (4) lack of protective effect from nucleophilic trapping agents and scavengers of reactive oxygen species against AOX1 inactivation (Fig. 6, D–I); (5) attenuation of the extent of AOX1 inactivation by varying concentrations of an alternative AOX substrate (*O*^6^-benzylguanine) and a competitive AOX1 inhibitor (gefitinib) (Fig. 7, Supplemental Figs 3 and 4); (6) presence of metabolism (inferred from Figs 4 and 8); and (7) molecular docking of the chemicals into the active site of AOX1, showing interactions with various amino acids within AOX1 active site (Fig. 5). Overall, these findings indicate that erlotinib, *O*-desmethylerlotinib, and *O*-didesmethylerlotinib bind to AOX1 active site, and the inactivating species remain within the active site prior to irreversible inactivation of AOX1.

Our inactivation kinetics analyses indicated that erlotinib and select metabolites inactivated AOX1 in a time- and concentration-dependent manner. As compared with erlotinib, *O*-desmethylerlotinib (removal of a methyl group at C_6_) has a higher *K*_I_, lower *k*_inact_/*K*_I_, higher partition ratio, whereas *O*-didesmethylerlotinib (removal of methyl groups at both C_6_ and C_7_) has a lower *K*_I_, higher *k*_inact_/*K*_I_, and comparable partition ratio. This indicates that the *O*-alkyl side chain at the C_6_ position of the quinazoline ring may enhance interactions with AOX1 and the loss of both *O*-alkyl side chain at the C_6_ and C_7_ positions may enhance interactions with AOX1, which were corroborated by our molecular docking experiments. In our study, the *K*_I_, *K*_I,u_, *k*_inact_, and *k*_inact_/*K*_I_ of erlotinib in AOX1 inactivation were 2.73 *μ*M, 1.52 *μ*M, 1.14 min^–1^, and 0.43 min^-1^*μ*M^–1^ (Table 1), respectively, which were in a similar range as the values (*K*_I_ = 0.72 *μ*M, *k*_inact_ = 0.36 min^–1^, and *k*_inact_/*K*_I_ = 0.50 min^–1^*μ*M^–1^) reported previously.^26^ The previous study did not calculate the *K*_I,u_ value. The slight differences in experimental values may be related to the different AOX substrates (carbazeran vs zoniporide) used in the experiments.

**Fig. 10:**
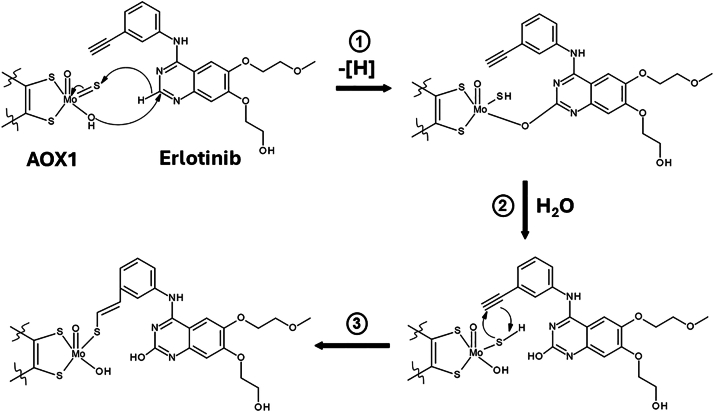
Proposed chemical mechanism of AOX1 inactivation by erlotinib. Nucleophilic attack at the C_2_ position of erlotinib (step 1); Mo(VI) cofactor in AOX1 is reduced in the catalysis and an OH-group is formed at the C_2_ position of erlotinib (step 2), and then a thiol-yne reaction between the alkyne group of erlotinib and the thiol group of Mo(IV) cofactor (reduced form) (step 3), thereby forming a covalent bond between erlotinib and AOX1.

Another major finding in this study is that the electron-rich terminal alkyne group of erlotinib, *O*-desmethylerlotinib, and *O*-didesmethylerlotinib is responsible for AOX1 inactivation. The terminal vinyl group in 3-vinylerlotinib attenuated the inactivation with an 11-fold higher potency (*K*_I_), 43-fold lower efficiency (*k*_inact_/*K*_I_ ratio), and a 10-fold higher partition ratio compared with erlotinib, whereas the terminal ethyl group in tetrahydroerlotinib (Supplemental Fig. 1) abolished the inactivation completely. This stark difference suggests that high electron density of the inactivator molecule (terminal acetylene and vinyl groups) contributes to greater potency and efficiency of AOX1 inactivation. Similar to tetrahydroerlotinib, gefitinib, *O*-desmethylgefitinib, and *O*-desmorpholinopropylgefitinib, which lack a terminal alkyne group (Supplemental Fig. 1), are not mechanism-based inactivators of AOX1, consistent with the notion that the alkyne group at C_4_ of the quinazoline ring (Supplemental Fig. 1) is responsible for AOX1 inactivation. Comparatively, hydralazine, a known AOX1 time-dependent inactivator,^10^ has a terminal hydrazine group which is also electron-rich. Consistent with our experimental data using structural analogs, our molecular docking analysis suggests that the interacting moiety is the alkyne group, and the covalent bond dissociation energy is lower for 3-vinylerlotinib. The formation of the covalent bond is through a typical thiol-yne reaction between the thiol group of the molybdenum cofactor and the alkyne group in erlotinib, which results in an alkenyl sulfide (-CH=CH-S-) metabolic product. Similarly, for 3-vinylerlotinib, a thiol-ene reaction results in the formation of a thioether (-C-S-C-). These findings provide insights into the covalent binding of AOX1 by erlotinib (Fig. 10, step 3). Therefore, based on our findings, it would be important to investigate whether other drugs with electron-rich terminal alkyne group and structural features of AOX1 substrate also inactivate AOX1. A recent study on the inactivation of AOX1 by another drug (icotinib^26^) with a terminal alkyne group supports our hypothesis. Further studies will be required to test the hypothesis with a broader range of drugs/chemicals. Of note, the oxidation of a C-C triple bond (acetylenic group) has also been associated with mechanism-based inactivation of specific cytochrome P450 enzymes,^40^ in contrast to the thio-yne reaction associated with the mechanism-based inactivation of erlotinib and select metabolites/structural analogs shown in the present study.

We probed further into the detailed mechanisms of inactivation by erlotinib using 4′-methylerlotinib (Supplemental Fig. 1), which is a structural analog of erlotinib. As compared with erlotinib, 4′-methylerlotinib inactivated AOX1 with a greater potency, but with a lesser inactivation rate, lower inactivation efficiency, longer inactivation t_1/2_, and greater partition ratio. A plausible explanation for the slightly higher inactivation potency observed for 4′-methylerlotinib may be the electron-donating methyl substituent increased electron density of the alkyne group, thereby favoring its covalent interaction with AOX1. Steric hindrance appears to affect inactivation rate more than it does the potency, as reflected by the lower *k*_inact_ and longer inactivation t_1/2_ for 4′-methylerlotinib compared with erlotinib, alluding to the possibility that inactivation rate is dependent on the size of inactivator molecule. This is similar to the findings that adding steric bulk even at a site remote from the vulnerable heterocycle can decrease susceptibility to AOX1 metabolism.^41^^,^^42^

Given that oxidation of erlotinib may occur at the electron-deficient C_2_ carbon of the quinazoline moiety, we used C_2_-substituted structural analogs to delve deeper into the mechanisms of inactivation by erlotinib. As compared with erlotinib, 2-methylerlotinib (Supplemental Fig. 1) inactivated AOX1 with a lesser potency, lower inactivation rate constant, lower inactivation efficiency, longer inactivation t_1/2_, and greater partition ratio, whereas 2-hydroxyerlotinib (Supplemental Fig. 1) inactivated AOX1 with a decrease in potency, lower inactivation rate constant, lower inactivation efficiency, longer inactivation t_1/2_, and greater partition ratio. The decreased potency and efficiency of 2-methylerlotinib and 2-hydroxyerlotinib suggest that the C_2_-position of erlotinib may play a role in the inactivation by erlotinib. These findings lead us to propose that erlotinib inactivation of AOX1 involves a nucleophilic attack at the C_2_ position of erlotinib, forming an OH-group at the C_2_ position (Fig. 10, steps 1 and 2). Moreover, changing the redox state with an exogenous oxidizing or reducing agent did not alter the extent of AOX1 inactivation by these structural analogs, suggesting that metabolism of the inactivator was needed to convert the Mo cofactor to the reduced state.

AOX is known to exhibit significant species differences in metabolism, enzyme kinetics, isoforms, and expression levels.^1^ However, as shown in the present study, species differences did not exist in the maximum extent of inactivation of human, rat, and mouse isoforms of AOX by erlotinib, suggesting that erlotinib was able to bind to the AOX in these species. Since the completion of our study, a study focusing on icotinib reported that erlotinib is a time-dependent inhibitor of human AOX1,^26^^,^^43^ consistent with the finding in the present study.

Our in vivo study in mice indicates that erlotinib increased the concentration of carbazeran (AOX probe substrate) and decreased the concentration of 4-oxo-carbazeran (metabolite) in blood, liver, and kidney. As shown in Fig. 9A, erlotinib increased the concentration of carbazeran (AOX probe substrate) in blood, liver, and kidney up to the last blood sampling time of 8 hours after dosing with carbazeran (ie, 10 hours after dosing with erlotinib). The elimination half-life of erlotinib has been reported to be 0.7 hours in mice after oral dosing with erlotinib.^39^ There should not be any erlotinib remaining in the systemic circulation in mice at 10 hours postdosing, ie, 14 half-lives. Therefore, competitive inhibition can be ruled out as a possible reason for the AOX1 inhibition evident at the 8-hour blood sampling time point; ie, 10 hours after erlotinib dosing. However, the carbazeran blood concentration-time profile after pretreatment with erlotinib would be consistent with mechanism-based inactivation of AOX1 in vivo in mice, where the inactivation effect is known to be prolonged.^44^

Our in vitro *K*_I,u_ value for erlotinib was 4-5-fold greater than the average in vivo plasma concentration of erlotinib (C_ss,u_) (Table 1). However, AOX1 accumulates substantively in the liver,^45^ and the level of erlotinib in the liver was approximately 3 times greater than that in the plasma.^46^ According to the US Food and Drug Administration guidelines for drug interaction studies,^47^ the risk of clinical interactions can be calculated based on these formulas: ratio = (k_obs_ + k_deg_) / k_deg_, where k_obs_ = (k_inact_ x 5 x C_max,u_) / (*K*_I,u_ + 5 x C_max,u_), and a risk ratio ≥ 1.25 requires further investigations using mechanistic static and/or physiologically based pharmacokinetic models. Due to the lack of studies on the first-order apparent rate of enzyme degradation (k_deg)_ for AOX1 in the literature, the average k_deg_ of 7 CYP enzymes (0.00033 min^–1^)^47^ was used. We obtained a ratio of 1761–1934 for erlotinib, 30–66 for desmethylerlotinib, and 2.5–8.3 for didesmethylerlotinib, which implies a high risk for clinical drug-drug interactions for erlotinib, *O*-desmethylerlotinib, and *O*-didesmethylerlotinib. In a clinical phase I study, erlotinib was shown to increase the plasma concentration of OSI-930,^48^ which was reported to be an AOX1 substrate.^49^ Overall, consistent with the in vitro inactivation of AOX1 by erlotinib, the administration of this drug to mice or humans impairs the in vivo elimination pharmacokinetics of an AOX-metabolized chemical.

In conclusion, erlotinib and select in vivo metabolites (*O*-desmethylerlotinib and *O*-didesmethylerlotinib) are mechanism-based inactivators of AOX1. Our novel findings lead us to propose that erlotinib inactivation of AOX1 involves a nucleophilic attack at the C_2_ position of erlotinib (inferred from Fig. 4, Fig. 8, Table 1 and Supplemental Fig. 5), reduction of the Mo(VI) cofactor in AOX1, formation of an OH-group at the C_2_ position of erlotinib, and a thiol-yne reaction between the alkyne group of erlotinib (Figs 3 and 5) and the thiol group of Mo(IV) cofactor (reduced form), thereby forming a covalent bond between erlotinib and AOX1 (Fig. 10). Overall, the present study provides mechanistic insights into the mechanism-based inactivation of AOX1 by erlotinib and the impact on the pharmacokinetics, safety, and efficacy of drugs and endogenous chemicals metabolized by AOX1.

## Conflict of interest

The authors declare no conflicts of interest.
