# Supplementary material for: Mechanism-based inactivation of human aldehyde oxidase by erlotinib: Mechanistic insights from structural analogs and molecular docking
Source: Mol Pharmacol. 2025 Nov 12;108(1):100097. doi: 10.1016/j.molpha.2025.100097 (PMC12881681; doi:10.1016/j.molpha.2025.100097)
Supplement: Supplementary Material 1 [file mmc1.pdf]

Mechanism-Based Inactivation of Human Aldehyde Oxidase by Erlotinib: Mechanistic Insights from Structural Analogues and Molecular Docking

Jia Rong Kweh [3], Nicholas Kai Ming Ng [3], Le Min Ngoh [3], Cynthia Jing Yan Li [3], Bao Jie Tan [3], Wee Kiat Tan [3], Vijaya Saradhi Mettu [4,a], Karl Austin-Muttitt [5], Jonathan G.L. Mullins [5], and Aik Jiang Lau [1, 2, *]

[1] *College of Pharmacy, Faculty of Health, Dalhousie University, Canada*

[2] *Department of Pharmacology, Faculty of Medicine, Dalhousie University, Canada*

[3] *Department of Pharmacy, Faculty of Science, National University of Singapore, Singapore*

[4] *Biological Resource Centre, Agency for Science, Technology and Research, Singapore*

[5] *Institute of Life Science, Swansea University Medical School, United Kingdom*

Figure Legends for Protein Data Bank (PDB) Files

PDB #1. Structure of human AOX1 protein with erlotinib

PDB #2. Structure of human AOX1 protein with *O*-desmethylerlotinib

PDB #3. Structure of human AOX1 protein with *O*-didesmethylerlotinib

PDB #4. Structure of human AOX1 protein with 3-vinylerlotinib

PDB #5. Structure of human AOX1 protein with hydralazine

PDB #6. Structure of human AOX1 protein

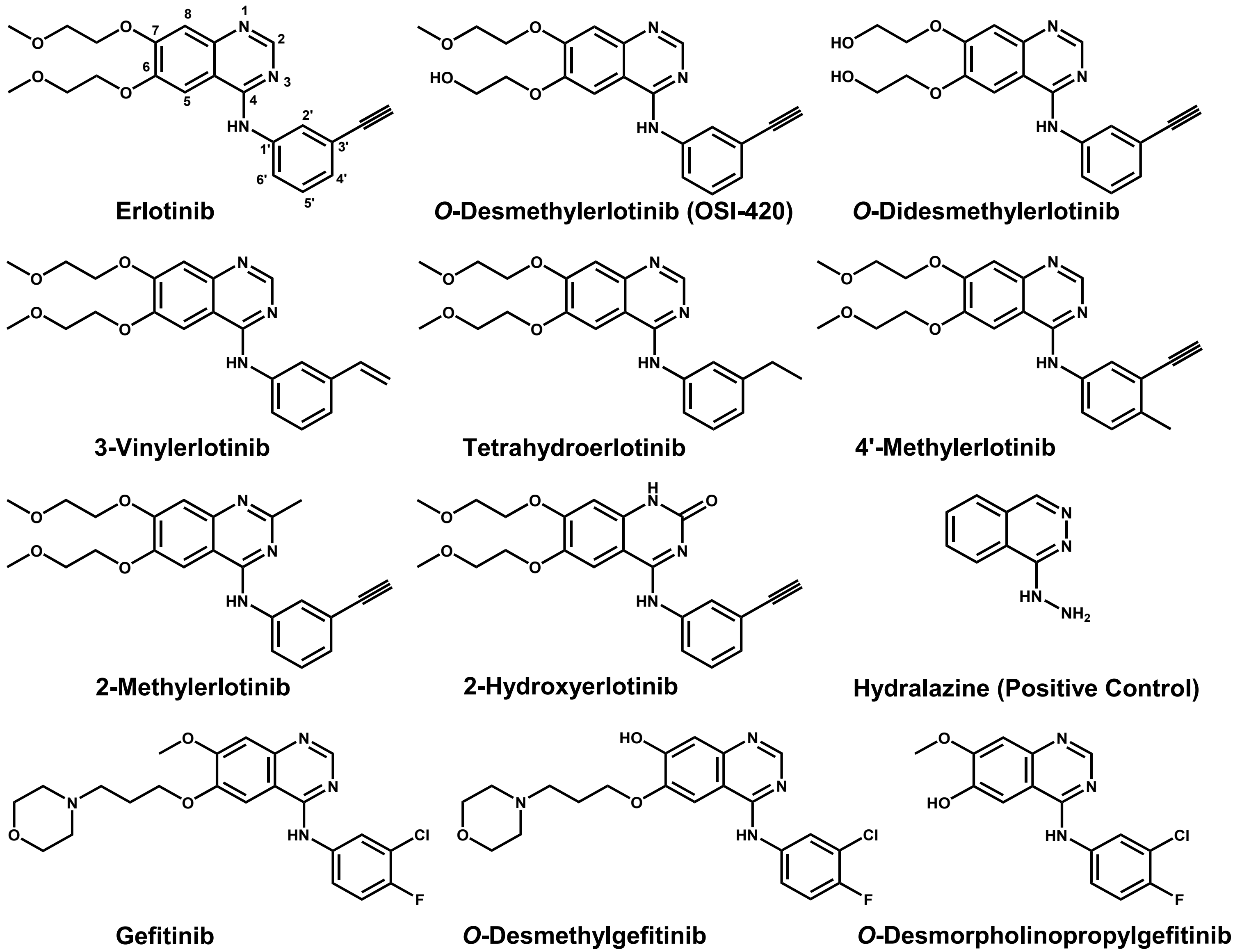

Supplemental Figure 1. Chemical structures of erlotinib, metabolites and structural analogues of erlotinib, gefitinib and select metabolites, and hydralazine (positive control).

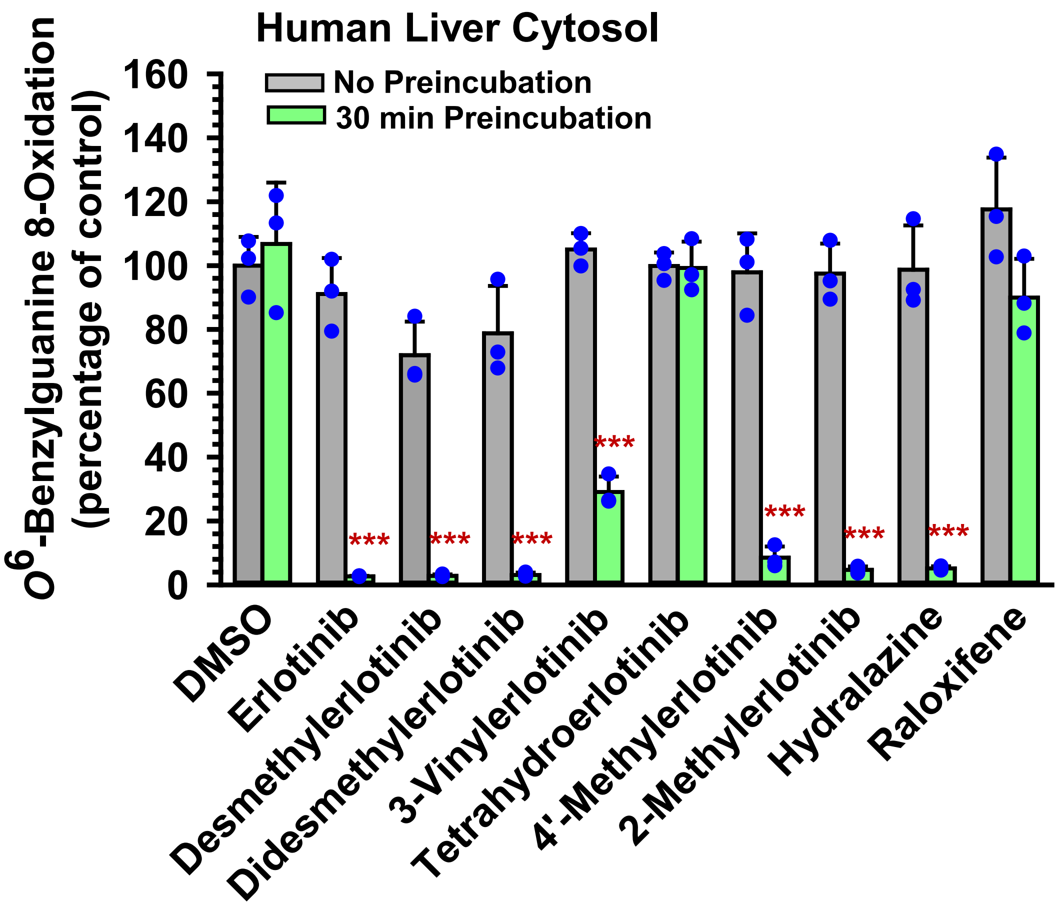

Supplemental Figure 2. Effect of preincubation of erlotinib, select metabolites and structural analogues with human liver cytosol on O^6-benzylguanine 8-oxidation. Human liver cytosol (100 µg protein) was preincubated with a test chemical (10 µM), hydralazine (10 µM; positive control), raloxifene (0.02 µM; negative control), or vehicle control (DMSO, 0.5% v/v) at 37°C for 0 or 30 min. An aliquot (10 µl) of the primary incubation mixture was transferred to a secondary incubation mixture containing O^6-benzylguanine (150 µM). Data are expressed as percentage of activity in the vehicle-treated control group that was not subjected to preincubation and expressed as mean ± S.D. of three independent experiments. $^{***}p < 0.001$, significantly different from 1) the vehicle-treated control group subjected to 30 min preincubation and 2) the same treatment group that was not subjected to preincubation.

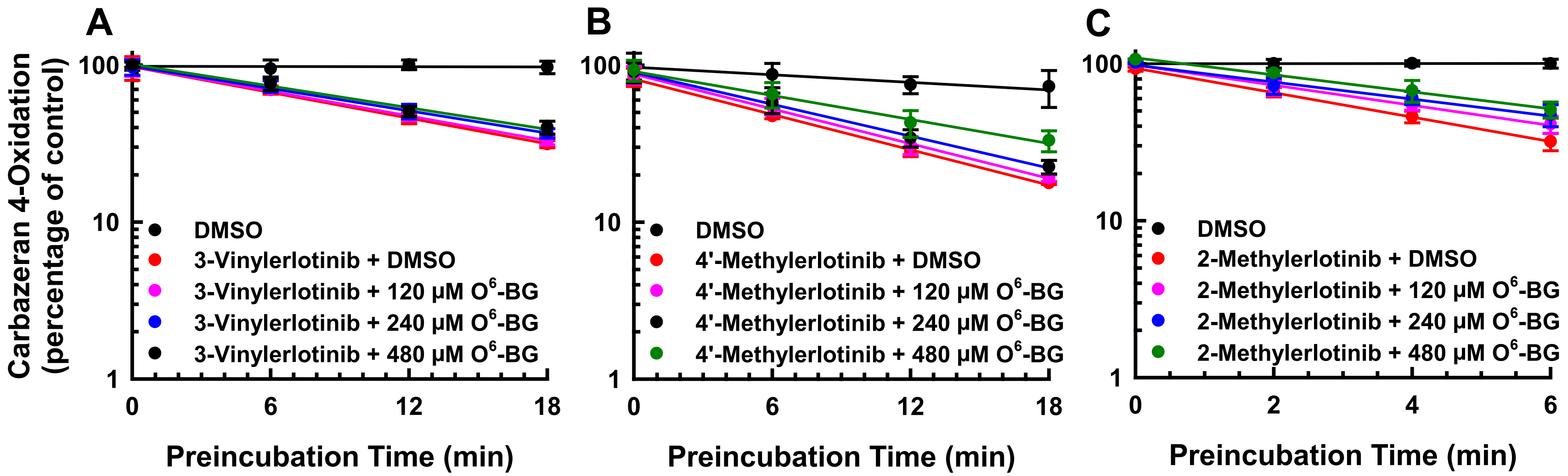

Supplemental Figure 3. Effect of an alternative AOX1 substrate (O^6-benzylguanine) on inactivation of human liver cytosolic carbazeran 4-oxidation by 3-vinylerlotinib, 4′-methylerlotinib, and 2-methylerlotinib. Human liver cytosol (100 µg protein) was preincubated with (A) 3-vinylerlotinib (10 µM), (B) 4′-methylerlotinib (3 µM), (C) 2-methylerlotinib (1 µM), or vehicle control (DMSO, 0.5% v/v) in the absence or presence of O^6-benzylguanine (120, 240, or 480 µM) at 37°C for (A, B) 0, 6, 12, or 18 min or (C) 0, 2, 4, or 6 min. An aliquot (10 µl) of the primary incubation mixture was transferred to a secondary incubation mixture containing carbazeran (15 µM). Data are expressed as percentage of activity in the vehicle-treated control group that was not subjected to preincubation and expressed as mean ± S.D. of three independent experiments.

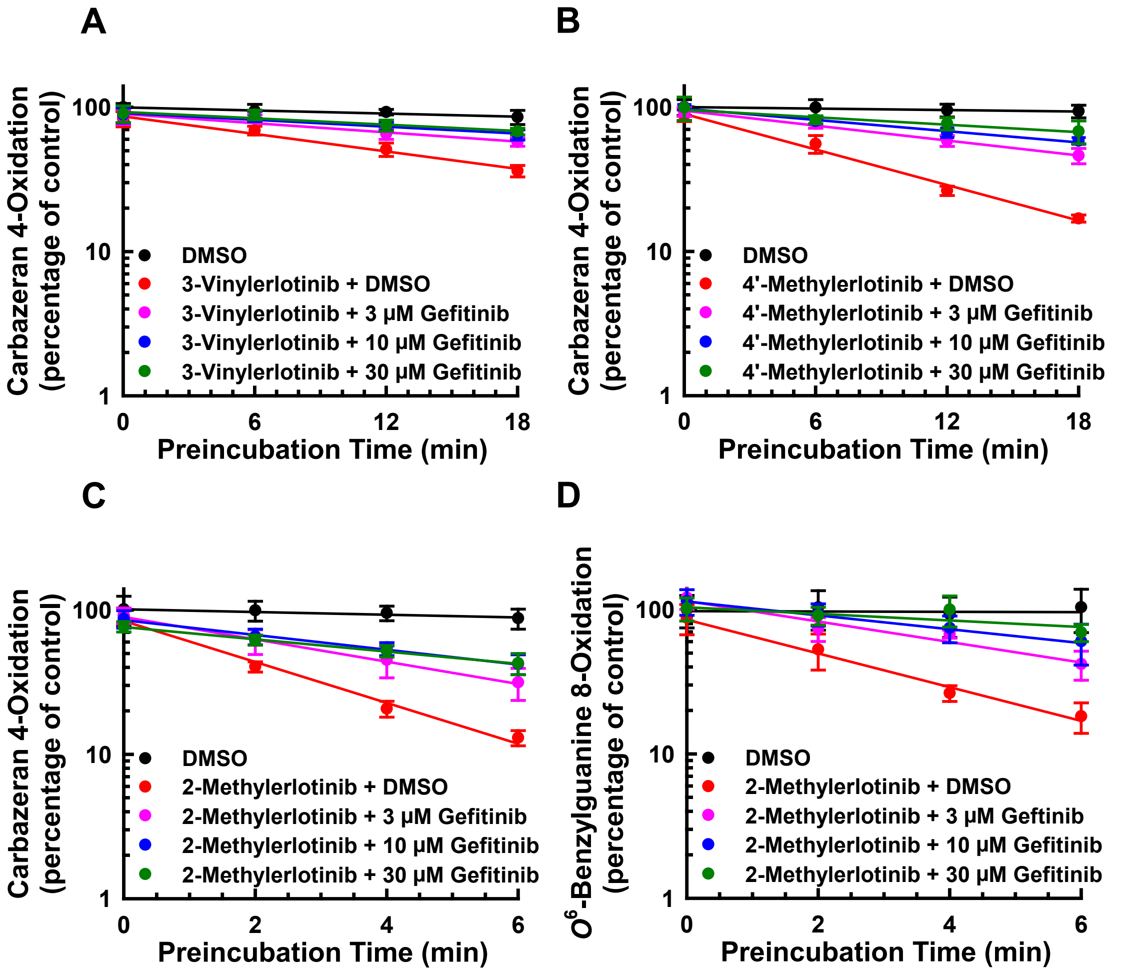

Supplemental Figure 4. Effect of a competitive AOX1 inhibitor (gefitinib) on inactivation of human liver cytosolic carbazeran 4-oxidation and O^6-benzylguanine 8-oxidation by 3-vinylerlotinib, 4′-methylerlotinib, and 2-methylerlotinib. Human liver cytosol (100 µg protein) was preincubated with (A) 3-vinylerlotinib (10 µM), (B) 4′-methylerlotinib (3 µM), (C, D) 2-methylerlotinib (2 µM), or vehicle control (DMSO, 0.5% v/v) in the absence or presence of gefitinib (3, 10, or 30 µM) at 37°C for (A, B) 0, 6, 12, or 18 min or (C, D) 0, 2, 4, or 6 min. An aliquot (10 µl) of the primary incubation mixture was transferred to a secondary incubation mixture containing carbazeran (15 µM) or O^6-benzylguanine (150 µM). Data are expressed as percentage of activity in the vehicle-treated control group that was not subjected to preincubation and expressed as mean ± S.D. of three independent experiments.

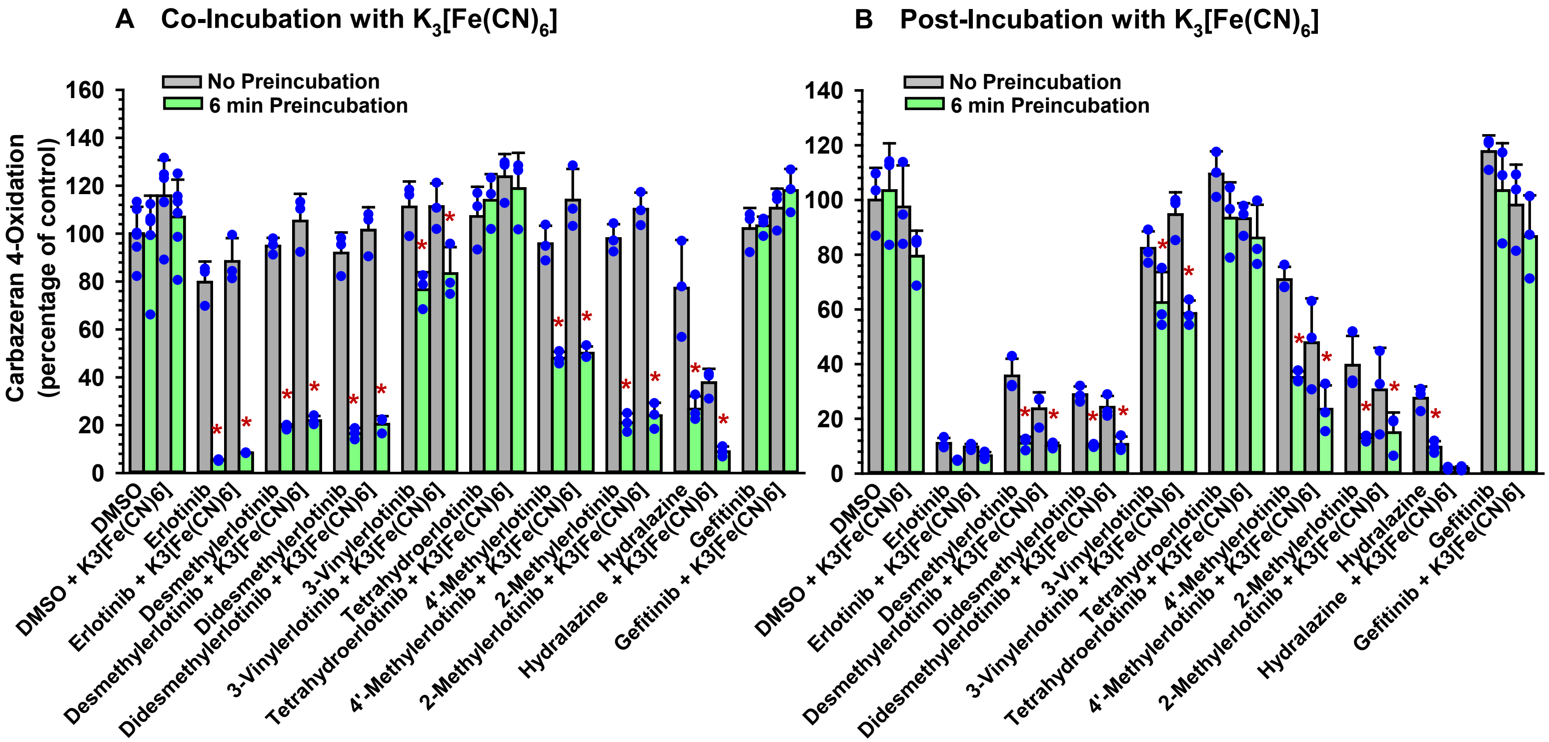

Supplemental Figure 5. Effect of an oxidizing agent on the inactivation of human liver cytosolic carbazeran 4-oxidation by erlotinib, select metabolites, and structural analogues. (A) Human liver cytosol (100 μg protein) was preincubated with a test chemical or vehicle control (DMSO, 0.5% v/v) in the absence or presence of potassium ferricyanide (2 mM) at 37°C for 0 or 6 min. An aliquot (10 μl) of the primary incubation mixture was transferred to a secondary incubation mixture containing carbazeran (15 μM). (B) Human liver cytosol (100 μg protein) was preincubated with a test chemical or vehicle control (DMSO, 0.5% v/v) at 37°C for 0 or 6 min. An aliquot (20 μl) of the primary incubation mixture was transferred to a secondary incubation mixture containing buffer or potassium ferricyanide (2 mM) at 37°C for 10 min, before another aliquot (50 μl) of the secondary incubation mixture was transferred to a tertiary incubation mixture containing carbazeran (15 μM). The test chemicals were erlotinib (1.5 μM), desmethylerlotinib (1.5 μM), didesmethylerlotinib (1.5 μM), 3-vinylerlotinib (10 μM), tetrahydroerlotinib (10 μM), 4′-methylerlotinib (3 μM), 2-methylerlotinib (2 μM), hydralazine (10 μM; positive control), and gefitinib (10 μM; negative control). Data are expressed as percentage of activity in the vehicle-treated control group that was not subjected to preincubation and expressed as mean ± S.D. of three independent experiments. *$p < 0.001$, significantly different from 1) the vehicle-treated control group subjected to 6 min preincubation and 2) the same treatment group that was not subjected to preincubation.

SUPPLEMENTAL TABLE 1

Optimized mass spectrometric parameters for the analysis of 3-vinylerlotinib, 4′-methylerlotinib, 2-methylerlotinib, and 2-hydroxyerlotinib by UPLC-MS/MS

Chemical	*m/z* transition (*m/z*)	Declustering potential (V)	Entrance potential (V)	Collision energy (V)	Collision cell exit potential (V)	Dwell time (ms)	Curtain gas (psi)	Collision-activated dissociation gas (psi)	Ion spray voltage (V)	Ion source temperature (°C)	Ion source gas 1 (psi)	Ion source gas 2 (psi)
Erlotinib Analogues												
3-Vinylerlotinib	396.2→280.1[a]	132.82	10	42.57	9.66	100	30	8	3500	650	40	40
	380.2→338.2			33.45	11.11							
4′-Methylerlotinib	407.6→292.2[a]	139.95	10	44.36	10.93	100	30	8	3500	650	40	40
	407.6→350.1			34.58	13.76							
2-Methylerlotinib	408.1→292.1[a]	166.56	10	44.84	10.13	100	30	8	3500	650	40	40
	408.1→350.2			34.21	12.30							
2-Hydroxyerlotinib	410.0→352.1[a]	109.98	10	24.49	12.96	100	30	8	3500	650	40	40
	410.0→118.1			37.75	10.07							

[a], *m/z* transition used for quantification.

SUPPLEMENTAL TABLE 2

Non-specific binding of erlotinib, select metabolites and structural analogues to human liver cytosol: Calculation of the unbound inhibition constants ($K_{i,u}$) for the inhibition of human liver cytosolic carbazeran 4-oxidation

A mixture containing a test chemical (10 µM; dissolved in a final concentration of 0.5% v/v DMSO) and human liver cytosol (100 µg total cytosolic protein) were dialyzed as described under *Materials and Methods*. Chemical concentration in each chamber was quantified by UPLC-MS/MS. Data are expressed as mean ± S.D. of three independent experiments.

Chemical	Fraction Unbound (f_u)	K_I (µM)	$K_{I,u}$ (µM)[a]
Erlotinib	0.56 ± 0.09	2.73 ± 0.73	1.52 ± 0.23
O-Desmethylerlotinib	0.84 ± 0.03	5.23 ± 1.24	4.41 ± 0.60
O-Didesmethylerlotinib	0.84 ± 0.03	1.98 ± 0.20	1.67 ± 0.10
3-Vinylerlotinib	0.78 ± 0.04	30.6 ± 9.60***	23.9 ± 4.34***
4′-Methylerlotinib	0.73 ± 0.05	1.03 ± 0.27	0.75 ± 0.12
2-Methylerlotinib	0.51 ± 0.07	4.00 ± 0.27	2.04 ± 0.08
2-Hydroxyerlotinib	0.78 ± 0.02	23.9 ± 3.68***	18.7 ± 2.87***

[a], $K_{I,u} = f_u \times K_I$, where f_u was calculated from 10 µM of chemicals in human liver cytosol.

***$p < 0.001$, significantly different from the parent drug (erlotinib) group.
